# Identification of the regulatory networks and hub genes controlling alfalfa floral pigmentation variation using RNA-sequencing analysis

**DOI:** 10.1186/s12870-020-2322-9

**Published:** 2020-03-12

**Authors:** Hui-Rong Duan, Li-Rong Wang, Guang-Xin Cui, Xue-Hui Zhou, Xiao-Rong Duan, Hong-Shan Yang

**Affiliations:** 1 Lanzhou Institute of Husbandry and Pharmaceutical Science, Chinese Academy of Agricultural Sciences, Lanzhou, China; 2 College of Ecological Environment and Resources, Qinghai Nationalities University, Xining, China; 3 Shanxi Electric Power Research Institute, State Grid Corporation of China, Taiyuan, China

**Keywords:** PacBio Iso-Seq, Transcriptome, Floral pigmentation, Alfalfa, Cream color, Hub gene

## Abstract

**Background:**

To understand the gene expression networks controlling flower color formation in alfalfa, flowers anthocyanins were identified using two materials with contrasting flower colors, namely Defu and Zhongtian No. 3, and transcriptome analyses of PacBio full-length sequencing combined with RNA sequencing were performed, across four flower developmental stages.

**Results:**

Malvidin and petunidin glycoside derivatives were the major anthocyanins in the flowers of Defu, which were lacking in the flowers of Zhongtian No. 3. The two transcriptomic datasets provided a comprehensive and systems-level view on the dynamic gene expression networks underpinning alfalfa flower color formation. By weighted gene coexpression network analyses, we identified candidate genes and hub genes from the modules closely related to floral developmental stages. *PAL*, *4CL*, *CHS*, *CHR*, *F3’H*, *DFR*, and *UFGT* were enriched in the important modules. Additionally, *PAL6*, *PAL9*, *4CL18*, *CHS2*, *4* and *8* were identified as hub genes. Thus, a hypothesis explaining the lack of purple color in the flower of Zhongtian No. 3 was proposed.

**Conclusions:**

These analyses identified a large number of potential key regulators controlling flower color pigmentation, thereby providing new insights into the molecular networks underlying alfalfa flower development.

## Background

Flower color is an important horticultural trait of higher plants [1]. Variation in flower color can fulfill an important ecological function by attracting pollinator’s visitation and influencing reproductive success in flowering plants [2], can protect the plant and its reproductive organs from UV damage, pests, and pathogens [3, 4], and has been of paramount importance in plant evolution [5, 6]. Furthermore, flower color is associated with the agronomic characters of plants directly or indirectly, and classical breeding methods have been extensively used to develop cultivars with flowers varying in color [7].

Three species of the genus *Medicago* L. are the most typical representatives of meadow ecosystems in the central part of European Russia: alfalfa (*M. sativa* L.), yellow lucerne (*M. falcata* L.), and black medic (*M. lupulina* L.), which are widely cultivated and grow easily in the wild [8–10]. The obvious differences in these species are their morphological features, among which flower color is the main trait used to distinguish them [11–13]. Understanding the differences in the growth period, botanical characteristics, agronomic characteristics, quality, and photosynthetic characteristics of different alfalfa germplasm materials associated with flower color would have great significance in alfalfa breeding [14, 15].

Of the above-mentioned *Medicago* species, purple-flowered alfalfa is the most productive perennial legume with high biomass productivity, an excellent nutritional profile, and adequate persistence [16, 17]. Yellow lucerne, which has yellow flowers, is closely related to alfalfa and exhibits better cold tolerance than alfalfa [18, 19]. Furthermore, the wild plants of *M. varia* with multiple flower color variations possess potential resistance to biotic and abiotic stressors [20]. The availability of abundant floral pigment mutants in *Medicago* species provides an ideal system for investigating the relationship between flower color and the stress resistance of alfalfa. Understanding the molecular mechanisms of flower color formation in alfalfa and identifying related key genes would contribute to the construction of an alfalfa core germplasm.

Flavonoids, carotenoids, and betalains are the three major floral pigments [21, 22]. Flavonoids, especially anthocyanidins, contribute to the pigmentation of flowers in plants [23, 24]. In the process of flower blooming, a somatic mutation from the recessive white to the pigmented revertant allele occurs, and flower variegation is inevitably the result of the differential expression of regulatory genes [25, 26]. To date, flower color-associated genes have been identified in many ornamental plants and in numerous studies, such as grape hyacinth, *Camellia nitidissima*, *Erysimum cheiri*, and *Matthiola incana* [27–29]. Using the crucial genes related to flower color formation to create new plant variety with special flower color, is circumvented by genetic engineering, while conventional breeding methods may be difficult to obtain the phenotype accurately [30]. For example, expression of the *F3’5’H* (flavonoid-3′, 5′-hydroxylase) gene in *Rosa hybrida* resulted in a transgenic rose variety with a novel bluish flower color not achieved by hybridization breeding [31]. By transferring antisense *CHS* (chalcone synthase) gene, a new petunia variety with white color was successfully obtained [32]. Although in many important ornamental crops, flower colors modification are already realized by molecular breeding, alfalfa varieties with special flower colors are often selected by natural selection for lacking the molecular mechanism of flower color formation.

RNA sequencing (RNA-Seq) technology has provided unique insights into the molecular characteristics of non-model organisms without a reference genome, and a series of genes involved in flavonoid pigment biosynthesis and carotenoid biosynthesis have been systematically analyzed [1, 33, 34]. However, the limitations of short-read sequencing lead to a number of computational challenges and hamper transcript reconstruction and the detection of splice events [35]. Chao et al. [36] found that, the PacBio Iso-Seq (isoform sequencing) platform could refine the data of short-read sequencing, including cataloging and quantifying transcripts and searching more alternatively spliced events.

Here, we used PacBio Iso-Seq combined RNA-Seq to identify specific genes related to flower color variation in two alfalfa materials with different flower colors. The dataset provides a comprehensive and system-level overview of the dynamic gene expression networks and their potential roles in controlling flower pigmentation. Using weighted gene coexpression network analysis (WGCNA), we identified modules of co-expressed genes and candidate hub genes for alfalfa with different flower colors. This work provides important insights into the molecular networks underlying alfalfa with cream flower pigmentation.

## Methods

### Plant material

High quality seeds of alfalfa cultivar Defu (C) were sent to the space by the “Shenzhou 3” recoverable spacecraft that flew in the space for 7 days (March 25th to 31th 2002). 1/3 of these space exposed seeds were planted alongside the control C in Xiguoyuan of Lanzhou city in 2009, a single plant with cream flower color was found and its seeds were collected individually. After planting the seeds in Qinwangchuan of Lanzhou city in 2010 isolatedly, 29 plants from the F1 generation possessed cream flower color. The seeds were collected, mixed and planted for another three generations, a mutant line with a cream flower color from F4 generation was confirmed in 2014. Compared to the control C, the mutant line exhibited stable cream flower color in the blooming period, which was named as “Zhongtian No. 3” (M). The original seeds of M were conserved in Lanzhou Institute of Husbandry and Pharmaceutical Science, Chinese Academy of Agricultural Sciences.

**Fig. 1:**
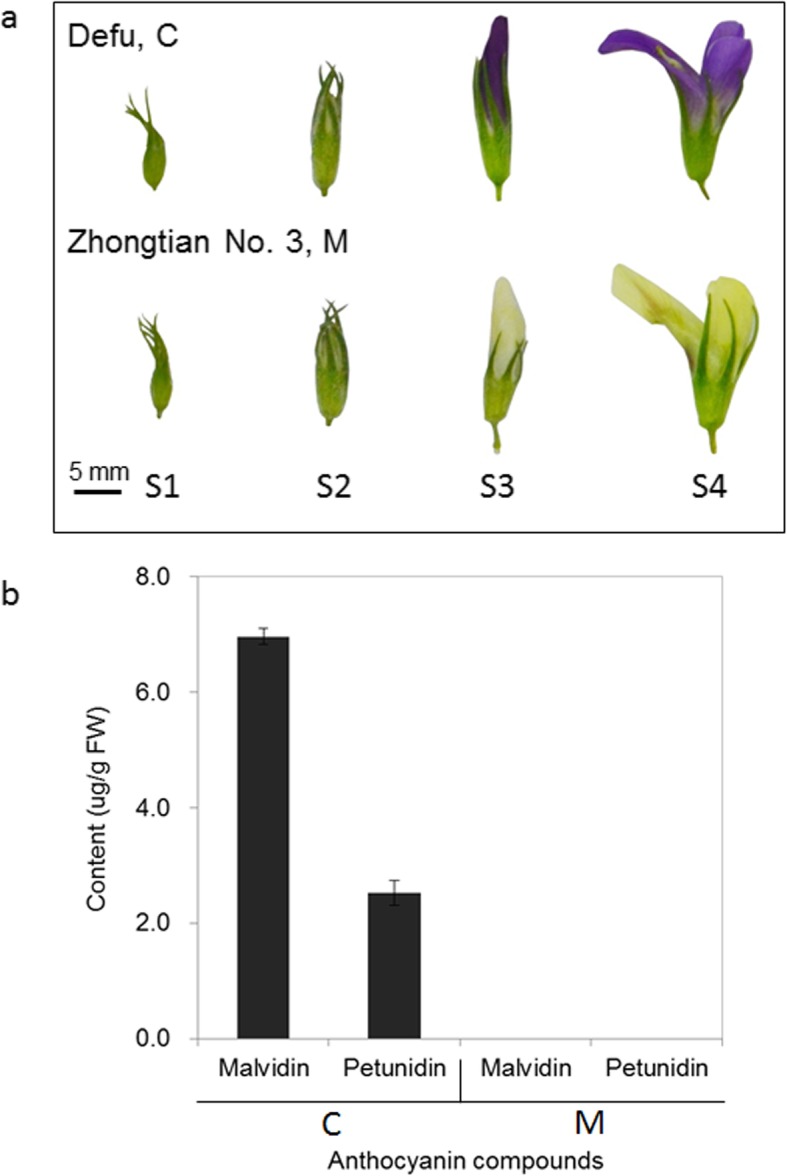
Phenotypes and anthocyanins compounds of the alfalfa materials. **a** Phenotypes of the different flower development stages from Defu and Zhongtian No. 3. **b** Anthocyanin compound contents in the peels of the two cultivars in S4. C, Defu; M, Zhongtian No. 3. Error bars indicate SEs

The alfalfa cultivar C and M were planted in the Dawashan experimental station (36°02′20′′ N, 103°44′36′′ E, 1697 H) of Lanzhou, Gansu, China in April 22th 2018. All seedlings of the same age were cultivated on homogenous loessal soil under the same management practices (soil management, irrigation, fertilization, and disease control). The petals of C and M were collected from four different development stages. The four stages were defined according to qualitative observations of the floral organs: S1 (the stage of the floret separating and the calyx packaging the petals), S2 (the stage of the petals appearing between the calyx lobes, with the length of the petals not exceeding more than 2 mm of the calyx), S3 (the stage where the petals exceed the calyx by 2 mm or more, the keel is still wrapped by the vexil, and during which the petals were just beginning to accumulate pigmentation), and S4 (the stage where the floret was in full bloom, with fully pigmented petals) (Fig. 1a). The four stages were assessed simultaneously for the indefinite inflorescence of alfalfa. Samples were harvested at the same time of day (9–11 AM) on July 4, 2018. Representative floral organs in each stage from three different plants were combined to form a sample, and three biological replicates were used for each floral development stage. All the samples in each stage endowed the same characteristics both of size and flower color, which were prepared for anthocyanin contents measurement and Illumina sequencing. Tissues of the leaves, shoots, stems, roots, flowers from the four different developmental stages above, and the young fruits from three C plants, were collected and pooled together in approximately equivalent weights. The mixed sample from 9 different tissues was then prepared for PacBio full-length sequencing. The samples were immediately frozen in liquid nitrogen and stored at − 80 °C until use.

### High-performance liquid chromatography analysis (HPLC) of anthocyanins

For anthocyanin extraction, fresh petal tissue was obtained from the fully-opened alfalfa flower in C-S4 and M-S4. Briefly, 0.5 g tissue from each sample was grounded in 1 mL of 98% methanol containing 1.6% formic acid at 4 °C. After 30 min of ultrasonic extraction, samples were centrifuged for 10 min at 12000 g, following with the supernatants were transferred to fresh tubes and the residual was extracted again. The supernatants were then combined and filtered through 0.45 mm nylon filters (Millipore). The standard substances included delphinidin 3-*O*-glucoside, cyanidin 3-*O*-glucoside, pelargonidin 3-*O*-glucoside, peonidin 3-*O*-glucoside, malvidin 3-*O*-glucoside, and petunidin 3-*O*-glucoside (ZZBIO Co., Ltd., Shanghai). According to the method of Tripathi et al. [24], 10 μL of the extract was analyzed using HPLC (Rigol L-3000, China). Mean values and standard errors (SEs) were obtained from three biological replicates.

### RNA quantification and assessment of quality

Total RNA was extracted using a mirVana miRNA Isolation Kit (Thermo Fisher Scientific, Waltham, MA, USA). RNA degradation and contamination were assessed on 1% agarose gels. The RNA quantity and quality were determined using a NanoDrop 2000 instrument (Thermo Fisher Scientific, Waltham, MA, USA), and RNA integrity was evaluated using an Agilent 2100 Bioanalyzer (Agilent Technologies, Santa Clara, CA, USA).

### PacBio Iso-Seq library preparation and sequencing

The sequencing library of 1 μg total RNA from the mixed sample of C was performed using the SMRTbell™ Template Prep Kit 1.0-SPv3 (Pacific Biosciences, Menlo Park, CA, USA). The amount and concentration of the final library was verified with a Qubit 2.0 Fluorometer (Life Technologies, Carlsbad, CA, USA). The size and purity of the library was determined using an Agilent 2100 Bioanalyzer (Agilent Technologies, Santa Clara, CA, USA). Following the Sequel Binding Kit 2.0 (Pacific Bioscience, USA) instruction for primer annealing and polymerase binding, the magbead-loaded SMRTbell template was performed on a PacBio Sequel instrument at Shanghai Oe Biotech Co., Ltd. (Shanghai, China).

### Illumina transcriptome library preparation and sequencing

The triplicate biological samples of two materials at the four stages yielded 24 non directional cDNA libraries (C-S1, C-S2, C-S3, C-S4, M-S1, M-S2, M-S3 and M-S4), which were obtained from 4 μg of total RNA. The size and purity of the libraries were tested with an Agilent 2100 bioanalyzer (Agilent Technologies, Santa Clara, CA, USA). The final libraries were generated using an Illumina HiSeq™ XTen instrument at Shanghai Oe biotech co., ltd. (Shanghai, China)

### PacBio data analysis

After the quality control of Isoseq (https://github.com/PacificBiosciences/IsoSeq_SA3nUP/wiki#datapub), including generation of circular consensus sequences (CCS), classification, and cluster analysis, high-quality consensus isoforms and low quality isoforms were recognized from the original subreads. Error correction of the high and low quality combined isoforms was conducted using the RNA-Seq data with the software LoRDEC. The corrected isoforms were compared with the reference genome using the software GMAP (http://www.molecularevolution.org/software/genomics/gmap). Afterward, redundant isoforms were then removed to generate a high-quality transcript dataset using the program TOFU (http://github.com/PacificBiosciences/cDNA_primer/) with an identify value of 0.85. The integrity of the transcript dataset was evaluated using the software BUSCO (v3.0.1) (https://busco.ezlab.org/). All identified non-redundant transcripts were searched by BLASTX (E-value ≤1^e-5^) against the protein databases of Non-redundant (NR), SWISS-PROT, and Kyoto Encyclopedia of Genes and Genomes (KEGG), and the putative coding sequences (CDS) were confirmed from the highest ranked proteins. Furthermore, the CDS of the unmatched transcripts were predicted by the package ESTScan. The non-redundant transcripts were compared to the PlantTFDB (http://planttfdb.cbi.pku.edu.cn/index.php) and the AnimalTFDB (http://bioinfo.life.hust.edu.cn/AnimalTFDB/) databases using BLAST to obtain the annotation information of the transcription factors (TFs).

The software AStalavista [37] was used to detect alternative splicing events in the sample. Transcripts with lengths greater than 200 bp were selected as lncRNA candidates, from which the open reading frames (ORFs) greater than 300 bp were filtered out. Putative protein-coding RNAs were filtered out using a minimum exon length and number threshold. LncRNAs were further screened using four computational approaches, including CPC2, CNCI, Pfam and PLEK.

### Illumina data analysis

Twenty-four independent cDNA libraries of flowers for C and M at different developmental stages were constructed according to a tag-based digital gene expression (DGE) system protocol. After removing low quality tags, including tags with unknown nucleotide “Ns”, empty tags, and tags with only one copy number, the clean tags were mapped to our transcriptome reference database. For the analysis of gene expression, the number of clean tags for each gene was calculated and normalized to FPKM (Fragments Per Kilobase of transcript per Million mapped reads). A *P*-value ≤0.05 in multiple tests and an absolute log_2_ fold change value ≥2 were used as thresholds for determining significant differences in gene expression.

### Weighted gene co-expression network analysis

The R package WGCNA was used to identify the modules of highly correlated genes based on the normalized expression matrix data [38]. The R package was used to filter the genes based on genes expression and variance (standard deviation ≤0.5). A total of 16,581 genes were ultimately remained. By conducting the function pickSoftThreshold, the soft threshold value of the correlation matrix was selected as 16, and the correlation coefficient was 0.83. The topological overlap (TO) matrix was generated by the TOM similarity algorithm, and then transcripts were hierarchically clustered with Hybrid Tree Cut algorithm 60 [29]. The first principal component was represented by the module eigengene.

### Real-time quantitative (RT-q) PCR validation

Twelve selected DEGs involved in flavonoid synthesis were determined by RT-qPCR. Total RNA was extracted from the 24 samples (in triplicate) as described above. First-strand cDNA was synthesized from 0.1 μg of total RNA by the manufacturer’s instruction (Vazyme, R223–01). The reactions were performed using a QuantiFast® SYBR® Green PCR Kit (Qiagen, Germany), and RT-qPCR was carried out on an Applied Biosystems QuantStudio™ 5 platform (Thermo Fisher Scientific, Waltham, MA, USA). The primers were designed with the Primer premier 5.0 software and synthesized by TsingKe Biological Technology Co., Ltd. (Xi’an, China) (Table S1). *Rer1* (JZ818481) was used as an internal standard [39]. The relative expression levels of genes were calculated using the 2^−ΔΔCt^ method [40].

### Statistical analysis

All RT-qPCR data were expressed as means ± SE (*n* = 3).

## Results

### Quantification of anthocyanidins

We quantified six anthocyanidins (delphinidin, cyanidin, pelargonidin, peonidin, malvidin, and petunidin) known to be involved in color development. Two high contents of malvidin and petunidin were detected in C-S4, the contents of which were 7.0 μg/g fresh weight (FW) and 2.5 μg/g FW, respectively. Otherwise, no color anthocyanidins were detected in the cream flowers of M-S4 (Fig. 1b).

### Sequencing and analysis of the floral transcriptome using the PacBio Iso-Seq platform

To identify transcripts that are as long as possible, the transcriptome of the mixed sample from different tissues of C (see Methods for details) were sequenced by the Iso-Seq system, yielding 14.33 million subreads. After the quality control of Isoseq, 140,995 isoforms were obtained, including 16,340 high-quality isoforms (accuracy > 99%). Most of the corrected isoforms (98.52%) were mapped to the *Medicago* genome (*M. truncatula* Mt4.0v2) using GMAP, and TOFU processing yielded 33,908 non-redundant isoforms (Table 1). The non-redundant transcript isoforms were used in subsequent analyses.

**Table 1:** PacBio Iso-Seq output statistics

Item	Total number	Total base (bp)	Min length	Max length	Mean length
Subreads	14328236	25008789438	50	106281	1745.419983
High quality isoforms	16340	33239138	336	8595	2034.218972
Low quality isoforms	124655	252521297	116	14650	2025.761478
Non-redundant isoforms	33899	72758476	156	14671	2146.331042

We compared the 33,908 isoforms against the *Medicago* genome set (Mt4.0v2), and 7784 (23%) new isoforms of annotated genes (ratio coverage < 50%) were obtained using MatchAnnot software (https://github.com/TomSkelly/MatchAnnot), and 513 novel isoforms were obtained that did not overlap with any annotated genes. To determine if the 513 novel isoforms were present in other plants, we conducted BLASTX searches against Swiss-Prot (E-value ≤ e^− 10^, see Methods). In total, 309 (60.23%) of these isoforms were annotated in the Swiss-Prot database, and the remaining isoforms were unannotated (Table S2).

**Fig. 2:**
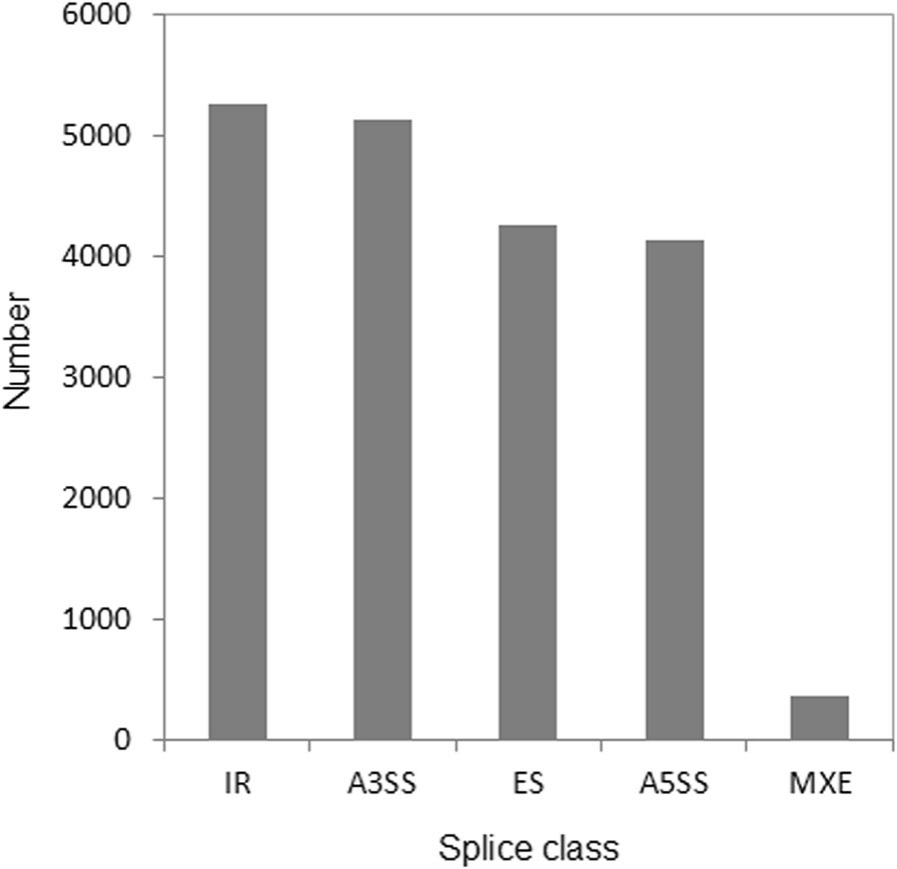
Alternative splicing events from the Iso-Seq. IR, intron retention. A3SS, alternative 3ˊ splice sites. ES, exon skipping/inclusion. A5SS, alternative 5ˊ splice sites. MXE, mutually exclusive exons

The numbers of isoforms distributed across the five main alternative splicing events were analyzed. IR (intron retention) was the most represented, accounting for 27.5% of alternative splicing transcripts (Fig. 2). MXE (mutually exclusive exons) were being the least, accounting for 1.9% of alternative splicing transcripts (Fig. 2).

By filtering and excluding transcripts with an ORF of more than 300 bp, 143 lncRNAs were finally obtained. The lncRNAs exhibited a wide length range from 202 bp to 2733 bp, and most of which (72%) were shorter than 700 bp. The average length of the lncRNAs (682 bp) was much shorter than the average length of all 33,908 isoforms (2146 bp).

### Sequencing and analysis of the floral transcriptome using the Illumina platform

For performance comparison and validation purposes, we also independently generated standard short read RNA-Seq data on the Illumina HiSeq™ XTen sequencing platform. Four floral organs from different developmental stages were sampled from both varieties. To this end, identification of DEGs from different floral organs could contribute to the understanding of the differential control of flower pigmentation. RNA-Seq analysis was performed on the samples described above with three biological replicates for each.

**Fig. 3:**
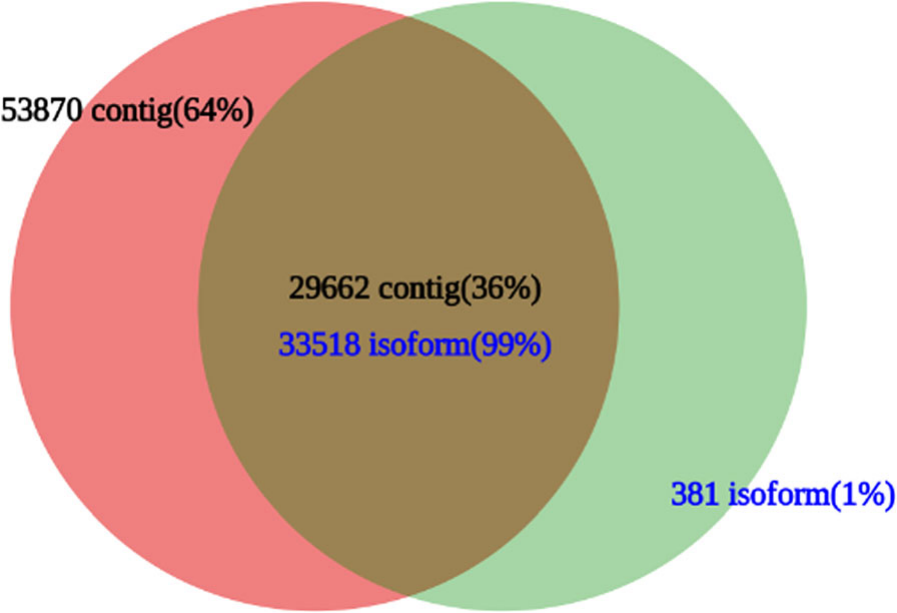
Comparison of isoforms from the PacBio Iso-Seq data and contigs from the RNA-Seq data

When compared to the PacBio transcript isoforms by BLASTN (coverage ≥0.85, e-value ≤1^e-20^, pairwise identity ≥90%, min bit score ≥ 100), 36% of the transcript contigs (29,662 contigs) exhibited similarity to 99% of the PacBio transcript isoforms (33,518 isoforms). There were 64% of the transcript contigs (53,870) and 1% of PacBio transcript isoforms (381 isoforms) that were unique to each of the datasets (Fig. 3).

**Fig. 4:**
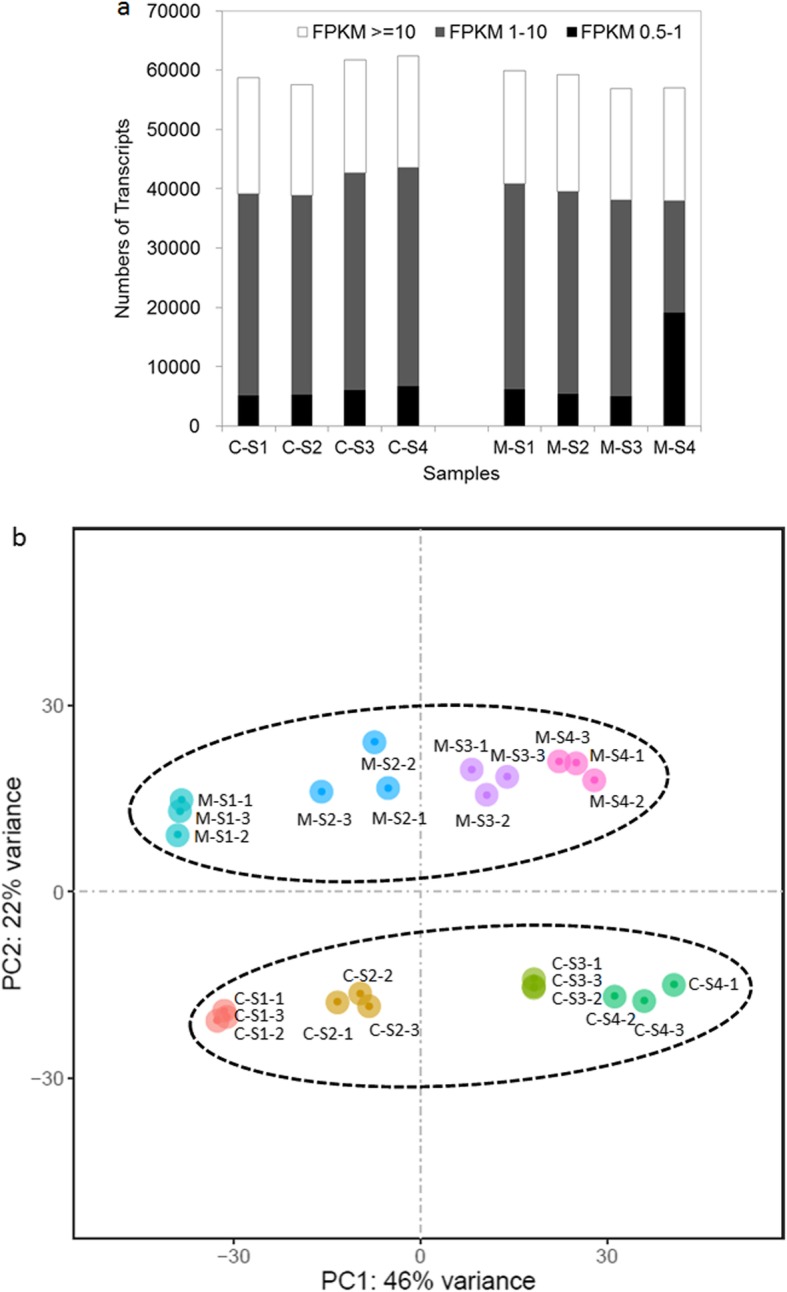
Global gene expression statistics in different floral development stages. **a** Numbers of detected transcripts in each sample. **b** Principal components analysis (PCA) of the RNA-Seq data

Transcripts with normalized reads lower than 0.5 FPKM were removed from the analysis. In total, 28,365, 28,242, 28,088, and 28,185 transcripts were found to be expressed in C-S1, C-S2, C-S3, and C-S4, respectively. Similarly, 27,810, 27,726, 27,711, and 27,878 transcripts were identified in the samples from the respective stages of M. The numbers of expressed transcripts distributed in the 0.5–1 FPKM range, 1–10 FPKM range and ≥ 10 FPKM range are indicated in Fig. 4a.

Principal component analysis (PCA) revealed that the 24 samples could be clearly assigned to eight groups as C-S1, C-S2, C-S3 C-S4, M-S1, M-S2, M-S3 and M-S4 (Fig. 4b). The samples of C and M from the same stage exhibited a distant clustering relationship, suggesting that the overall transcriptome profile is evidently different for C and M at each developmental stage (Fig. 4b).

### DEGs during the flower developments of alfalfa materials with purple and cream flower

**Fig. 5:**
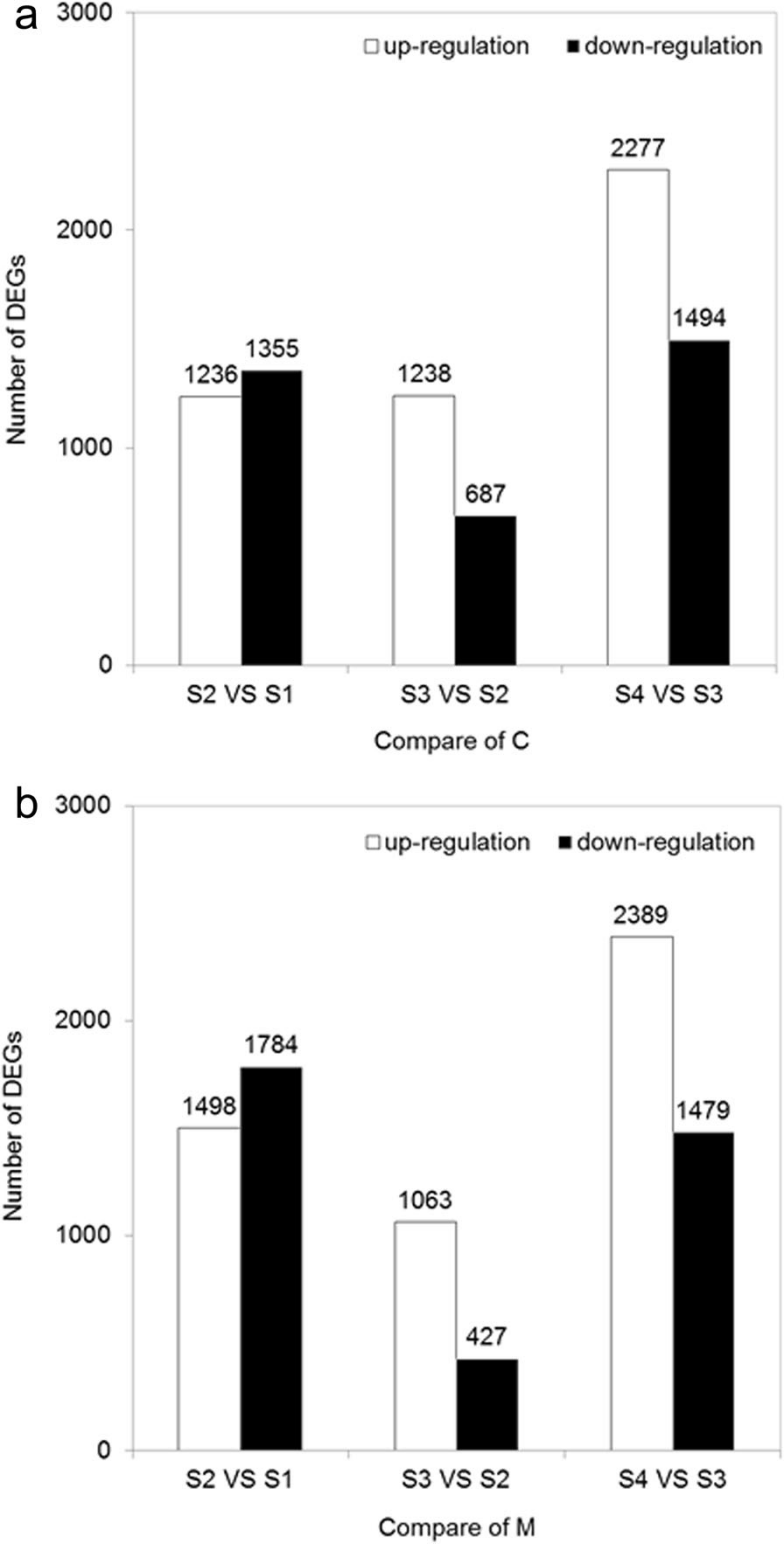
Number of DEGs between the different floral development stages. **a** DEGs of alfalfa cultivar C. **b** DEGs of alfalfa cultivar M. C, Defu; M, Zhongtian No. 3

The differences in gene expression were analyzed by comparing the four different floral development stages, using the thresholds of false discovery rate (FDR) value < 0.05 and fold change > 2. In total, 2591, 1925 and 3771 DEGs were identified between C-S2 vs C-S1, C-S3 vs C-S2, C-S4 vs C-S3, respectively (Fig. 5a). Similarly, 3282, 1490 and 3868 DEGs were identified between M-S2 vs M-S1, M-S3 vs M-S2, M-S4 vs M-S3, respectively (Fig. 5b). Contrasting S2 with S1, the down-regulated unigenes of C and M were similar to the up-regulated unigenes. Differently, the up-regulated unigenes were dominant between S3 vs S2, as well as between S4 vs S3 in both C and M.

**Fig. 6:**
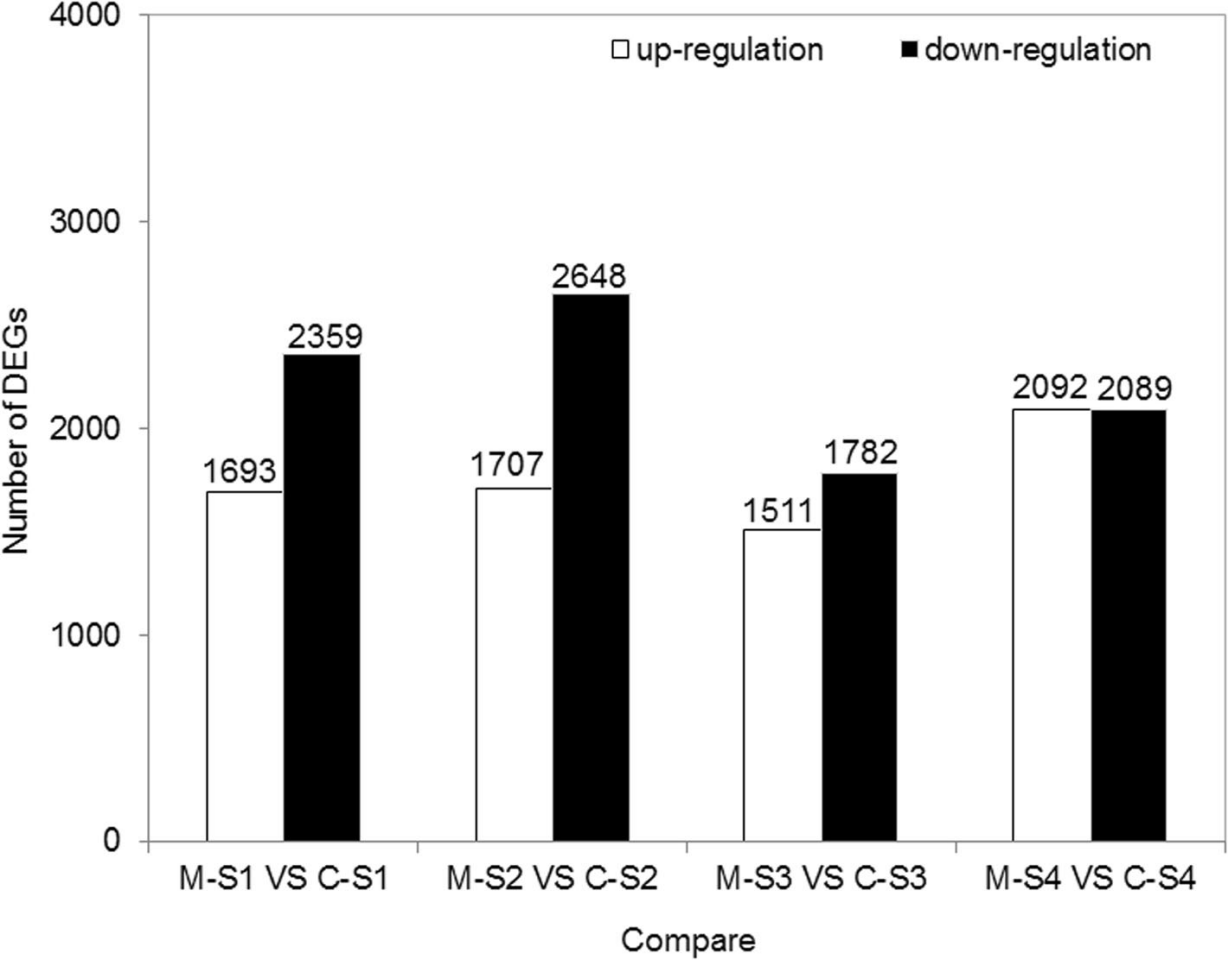
Comparison of the DEGs between the two cultivars. C, Defu; M, Zhongtian No. 3

In order to analyze the flower color formation differences in C and M, we compared the DEGs of C and M in the same flower development stage. In total, 4052, 4355, 3293, and 4181 DEGs were identified between M-S1 vs C-S1, M-S2 vs C-S2, M-S3 vs C-S3, and M-S4 vs C-S4, respectively. Furthermore, 1693, 1707, 1511, and 2092 DEGs were up-regulated, respectively (Fig. 6).

To identify the metabolic pathways related to flavonoid biosynthesis that were enriched, an analysis of KEGG pathway was conducted by comparing different flowering stages in C and M. With the flower blooming, the enriched pathways related to flavonoid biosynthesis increased evidently. Especially, between M-S4 vs C-S4, flavone and flavonol biosynthesis (ko00944), flavonoid biosynthesis (ko00941) and phenylpropanoid biosynthesis (ko00940) were enriched on the top 5 KEGG pathways (Figure S1), implying the crucial flower color formation stage.

### Transcriptional profiles of the genes related to flavonoid biosynthesis

**Fig. 7:**
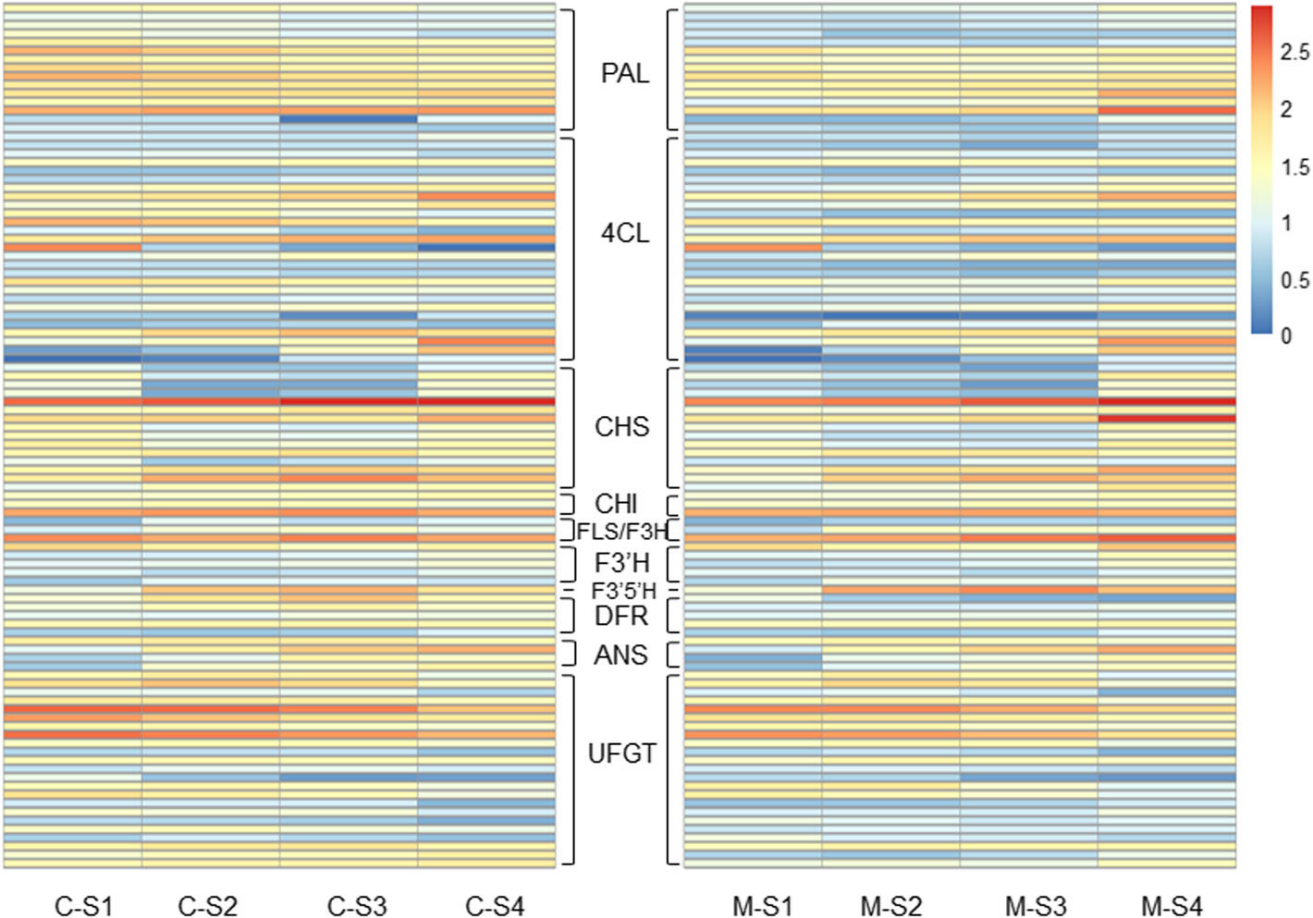
Expression heatmap of the DEGs of flavonoid biosynthesis. The expression of DEGs is displayed as log_10_ (FPKM+ 1). *PAL*, phenylalanine ammonia-lyase; *4CL*, 4-coumarate: coenzyme A ligase; *CHS*, chalcone synthase; *CHI*, chalcone isomerase; *FLS*, flavonol synthesis; *F3H*, flavanone 3-hydroxylase; *F3′H*, flavonoid 3′-hydroxylase; *F3′5′H*, flavonoid 3′5′-hydroxylase; *DFR*, dihydroflavonol 4-reductase; *ANS*, anthocyanidin synthase; *UFGT*, UDP-glucose: flavonoid 3-O-glucosyltransferase

To determine the key genes involved in flavonoid biosynthesis, the genes with FPKM values lower than 5 were excluded. Phenylalanine ammonia-lyase (*PAL*, 15 isoforms), 4-coumarate: coenzyme A ligase (*4CL*, 27 isoforms), *CHS* (15 isoforms), chalcone isomerase (*CHI*, 3 isoforms), flavanone 3-hydroxylase (*F3H*) / flavonol synthesis (*FLS*) (3 isoforms), flavonoid 3′-monooxygenase (*F3′H*, 5 isoforms), *F3′5′H* (1 isoform), dihydroflavonol 4-reductase (*DFR*, 5 isoforms), anthocyanidin synthase (*ANS*, 4 isoforms), and UDP-glucose: flavonoid 3-O-glucosyltransferase (*UFGT*, 23 isoforms) were identified (Table S3). The expression pattern of the total of 101 isoforms (encoding 11 enzymes) was displayed in the heatmap, and the isoforms showed different changes during flower development in both C and M (Fig. 7).

Among these DEGs, most *PAL* genes showed down-regulated expression changes in C, but up-regulated expression patterns in M. In general, the FPKM values of many *PALs* were significantly higher in C than M (Fig. 7). It is possible that these PALs may be crucial in the formation of flower colors. Most genes encoding *4CLs*, *CHSs*, *CHIs*, *FLS*/*F3Hs*, *F3’Hs*, *F3’5’Hs*, *ANSs*, and *UFGTs* exhibited similar expression patterns in both C and M with flower blooming However, the FPKM values differed greatly between C and M, indicating differential expression abundance in C and M. Additionally, we found 4 *DFRs* with different expression changes in C and M (particularly *DFR1* and *DFR2*), the FPKM values of which were evidently higher in C than M, implying their potential functions in color formation in different flowers (Fig. 7).

### Gene co-expression network analysis based on flower pigments

**Fig. 8:**
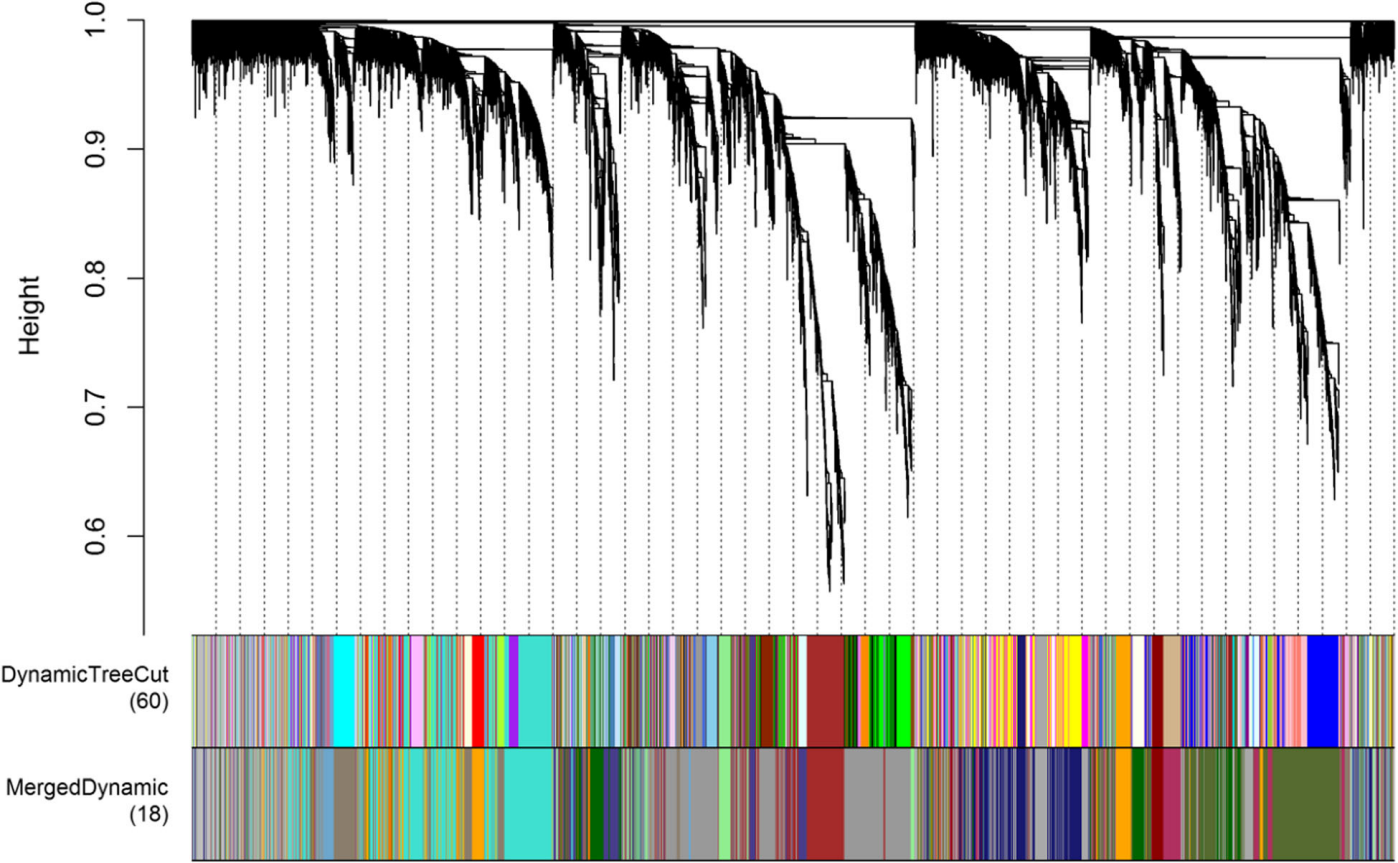
Gene co-expression modules detected by WGCNA. The clustering dendrogram of the genes across all the samples exhibits dissimilarity based on topological overlap, together with the original module colors (dynamic tree cut) and assigned merged module colors (merged dynamic)

**Fig. 9:**
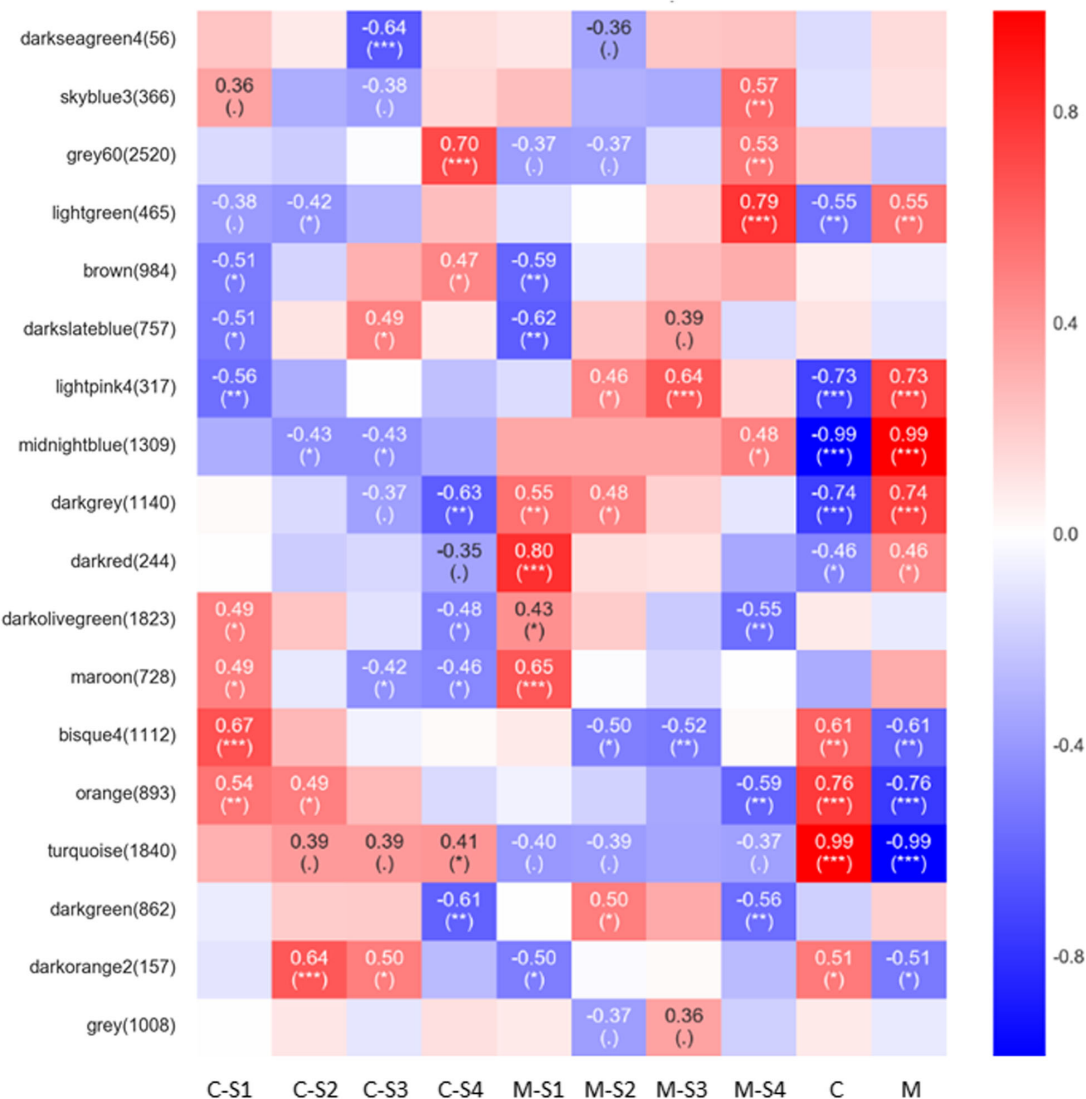
Module-trait associations using WGCNA. Each row corresponds to a module eigengene and each column to a stage. Each cell contains the corresponding correlation and *P*-value. The table is color-coded by correlation, according to the color legend

To reveal the regulatory network correlated with the changes in the successive developmental stages across the two varieties, we constructed the co-expression modules analysis by WGCNA (Fig. 8). Co-expression networks were constructed on the basis of pairwise correlations of gene expression across all samples. Modules were defined as clusters of highly interconnected genes, and genes within the same cluster have high correlation coefficients among them. From WGCNA, 18 co-expression modules were constructed, of which, the grey 60 module was the largest module, consisting of 2520 unigenes, whereas the darkseagreen 4 module was the smallest, consisting of only 56 unigenes. The distribution of isoforms in each module (labeled with different colors) and module-trait correlation relationships is shown in Fig. 9. A number of modules displayed a close relationship with different stages.

The most important modules of our concern were the modules enriched in the C or M group, especially in S4 of C and M, which could help to distinguish the flower color phenotype. The modules of interest were thus selected according to the criteria |r| > 0.5 and *P* < 0.05, and were further annotated by KEGG and GO analysis. The module of skyblue 3 displayed a close relationship with M-S4. In the skyblue 3 module, many pathways related to color formation were enriched (*P* < 0.01). Among them, flavonoid biosynthesis (ko00941) and phenylpropanoid biosynthesis (ko00940) were the top 2 pathways (Table S4). Furthermore, the modules of bisque 4 and turquoise exhibited a close relationship with M or C, the enriched pathways (*P* < 0.01) of which were summarized in Table S4.

### Candidates responsible for the loss of purple color in alfalfa with cream-colored flower

The expression patterns of 23 candidate genes according to the closed modules are indicated in Table 2. In summary, all 9 *PALs* were down-regulated during the flower ripening process in C, while in M-S4, they remained stable or declined initially and then increased. Additionally, their relative expression levels in S1-S3 of C were significantly higher than in M. Importantly, *PAL6* and *PAL9* were identified as candidate hub genes for the module of bisque 4. *4CL18* and *4CL22* were enriched in the module of bisque 4, and *4CL18* was identified as a candidate hub gene for this module. The much higher expression levels of *4CL18* in S1-S3 of C, which were evidently higher than M, were suggestive of a particularly important role for 4CL18 in the pathway. Four *CHSs* were enriched in the module of skyblue 3, in which, *CHS2*, *CHS4*, and *CHS8* were identified as candidate hub genes. They possessed the same expression changes in different stages of C and M, and in the M-S4, the relative expression levels of *CHS2*, *CHS4*, and *CHS8* were 2.1-, 1.3-, and 2.5-fold higher than in C-S4. We also searched 3 *CHRs* enriched in these important modules, and found that the expression change patterns of *CHR1*, *CHR2*, and *CHR3* were consistent with the enriched *CHSs*. Furthermore, *F3’H4*, *DFR1*, *DFR2*, *UFGT22*, and *UFGT23* were enriched in these modules. In S1 and S2, the expression levels of *F3’H4* were 1.2- and 2.0- fold higher in C than in M. With flower development in C, *DFR1* was up-regulated and peaked at S3, however, *DFR1* exhibited almost no expression in M. *DFR2* was up-regulated and peaked at S3 in C, however, it exhibited low expression abundance and remained stable in M. The expression levels of *DFR1* and *DFR2* were evidently higher in all of the stages of C than M. Higher expression levels in C were also found in *UFGT22* and *UFGT23* (Table 2).

**Table 2:** FPKM value statistics of 23 candidate genes in the closed modules

Gene name	Isoform ID	FPKM value							
		C-S1	C-S2	C-S3	C-S4	M-S1	M-S2	M-S3	M-S4
PAL1	PB.11849.10|chr7:40942885–40960253(+)|i2_LQ_samplef2cfa8|c97668/f1p0/2837	35.55	35.96	23.56	14.21	13.01	13.55	13.41	27.42
PAL2	PB.11849.12|chr7:40942885–40959874(+)|i2_LQ_samplef2cfa8|c71112/f1p0/2392	11.03	12.11	7.05	7.75	6.82	5.44	7.74	12.71
PAL3	PB.11849.14|chr7:40942885–40960512(+)|i3_LQ_samplef2cfa8|c5006/f1p3/3081	15.97	14.52	9.46	10.16	7.08	5.73	7.73	12.41
PAL4	PB.11849.16|chr7:40942887–40959833(+)|i2_LQ_samplef2cfa8|c94554/f1p1/2514	21.12	12.71	8.35	4.72	7.52	2.90	4.04	3.68
PAL6	PB.11849.2|chr7:40942885–40959392(+)|i1_LQ_samplef2cfa8|c131710/f1p63/1911	153.74	108.77	68.88	52.63	89.36	40.94	35.96	53.15
PAL7	PB.11849.3|chr7:40942885–40959926(+)|i2_HQ_samplef2cfa8|c113826/f130p0/2449	43.81	38.00	33.44	24.79	26.81	24.34	26.89	49.16
PAL8	PB.11849.4|chr7:40942885–40945992(+)|i2_LQ_samplef2cfa8|c4595/f1p3/2499	92.78	61.43	41.47	35.18	52.91	30.09	20.42	28.56
PAL9	PB.11849.8|chr7:40942885–40959918(+)|i2_LQ_samplef2cfa8|c27489/f1p1/2504	149.34	116.95	84.52	61.45	99.89	56.62	49.55	91.97
PAL15	PB.9841.1|chr5:43212802–43217702(−)|i2_HQ_samplef2cfa8|c6525/f8p0/2317	7.40	6.06	4.26	2.93	6.86	4.36	3.01	4.45
4CL18	PB.5838.1|chr4:349590–353192(+)|i1_HQ_samplef2cfa8|c237238/f40p8/1909	83.07	62.39	41.54	28.99	30.55	16.48	15.56	51.90
4CL22	PB.8087.5|chr4:53453111–53459491(+)|i1_LQ_samplef2cfa8|c10179/f1p0/1987	3.41	3.27	0.51	5.77	0.46	0.20	0.36	1.02
CHS1	PB.10727.1|chr7:5288756–5290374(−)|i1_LQ_samplef2cfa8|c23258/f3p20/1452	10.24	2.46	2.49	8.30	4.75	2.61	1.14	8.10
CHS2	PB.10728.1|chr7:5301940–5316126(+)|i1_LQ_samplef2cfa8|c190118/f2p13/1386	34.31	6.70	4.45	31.23	15.62	6.70	4.04	66.58
CHS4	PB.10728.3|chr7:5301944–5316192(+)|i1_HQ_samplef2cfa8|c217277/f2p10/1333	15.81	1.35	2.35	18.09	9.13	3.34	2.27	23.23
CHS8	PB.1696.1|chr1:44128070–44142309(+)|i1_LQ_samplef2cfa8|c11658/f1p17/1523	35.79	9.14	7.26	22.11	21.65	8.89	6.83	55.98
CHR1	PB.9832.2|chr5:42889648–42891090(+)|i1_LQ_samplef2cfa8|c117495/f1p6/1258	22.31	5.33	3.37	24.62	20.12	6.06	3.31	50.52
CHR2	PB.9833.6|chr5:42874302–42875653(−)|i1_LQ_samplef2cfa8|c203391/f1p6/1151	6.33	1.70	1.97	6.82	5.77	2.06	1.81	21.22
CHR3	PB.9833.7|chr5:42883325–42884800(−)|i1_LQ_samplef2cfa8|c126525/f1p6/1270	14.73	2.13	3.79	9.10	10.79	2.13	0.68	30.23
F3’H4	PB.7478.2|chr4:42392721–42394930(−)|i1_HQ_samplef2cfa8|c1984/f8p1/1981	9.37	4.63	5.21	10.27	11.26	9.17	4.75	10.66
DFR1	PB.339.2|chr1:7156508–7160534(−)|i1_HQ_samplef2cfa8|c21297/f2p0/1255	18.91	75.03	123.51	36.44	16.53	3.76	1.94	1.33
DFR2	PB.340.1|chr1:7164081–7167125(−)|i1_LQ_samplef2cfa8|c4738/f1p0/1273	15.51	31.71	45.29	22.74	9.27	7.72	8.19	15.33
UFGT22	PB.11876.1|chr7:41535946–41537368(+)|i1_HQ_samplef2cfa8|c67068/f2p2/1422	19.33	29.03	29.83	50.50	4.40	3.04	4.97	14.76
UFGT23	PB.11878.1|chr7:41563371–41564959(+)|i1_LQ_samplef2cfa8|c116694/f1p0/1538	32.07	41.14	42.39	51.69	16.29	18.70	15.55	34.02

**Fig. 10:**
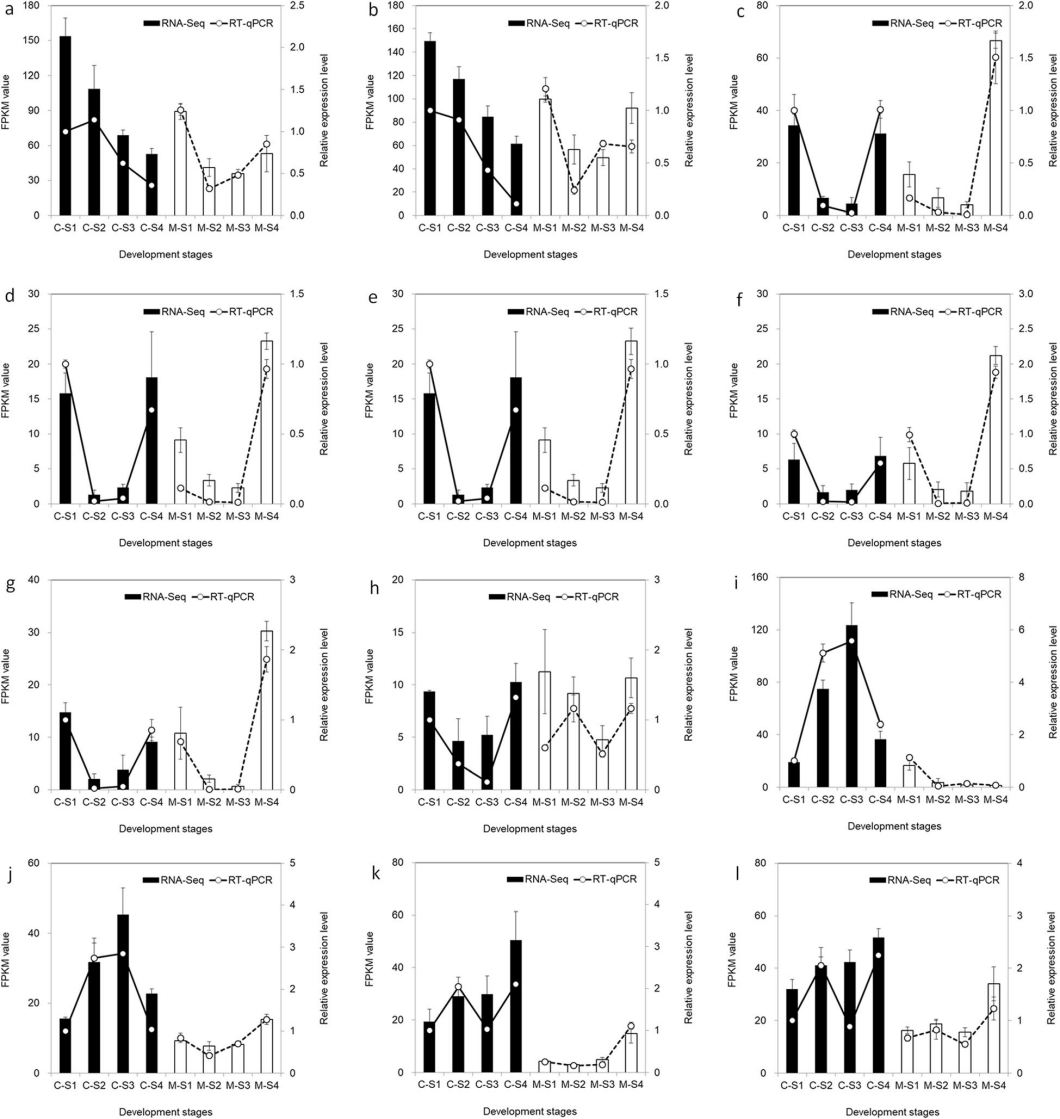
Expression profiles of 12 candidate genes and RT-qPCR validation. *EF1a* is used as the internal control. The error bars represent the SEs of the RT-qPCR data (*n* = 3). “r” represents the Pearson correlation coefficient. Pearson’s correlations between the RNA-Seq data and RT-qPCR data are calculated using the log_2_ fold change and the relative expression level. **a***PAL6*; **b***PAL9*; **c***CHS2*; **d***CHS4*; **e***CHR1*; **f***CHR2*; **g***CHR3*; **h***F3’H4*; **i***DFR1*; **j***DFR2*; **k***UFGT22*; **l***UFGT23*

To further confirm these results and verify the expression of the above genes in the C and M, RT-qPCR was performed to analyze the expression patterns of 12 genes (Fig. 10). Most genes exhibited similar expression patterns between the RT-qPCR and RNA-Seq data, which confirmed the reliability of the RNA-Seq data.

## Discussion

### Anthocyanin identification from the peels of two different materials

Color mutants are widely used in horticultural and other crops, especially those that are commonly propagated vegetatively, such as most fruit trees [41, 42]. Purple color in the flower petals of alfalfa (*M. sativa* L., *M. falcata* L. and their hybrids) is due to the presence of sap-soluble anthocyanins [43]. The floral anthocyanins of alfalfa have been widely studied. Lesins [44] identified alfalfa flower with three pigments as glycosides of petunidin, malvidin and delphinidin. Furthermore, Cooper and Elliott [45] identified alfalfa flower with three anthocyanins as 3,5-diglucosides of petunidin, malvidin and delphinidin. Differently, using HPLC, we only found that malvidin 3-*O*-glucoside and petunidin 3-*O*-glucoside in the purple flower of C, while no color pigment was detectable in the cream flowers of M (Fig. 1). The results suggest that the drastic differences in anthocyanin accumulation are a result of cultivar and genetic specificity.

### PacBio full-length sequencing extends the alfalfa annotation and increases the accuracy of transcript quantification

Due to technical limitations, the reference genome of alfalfa is not presently available. Our current knowledge on the alfalfa transcriptome is mainly based on RNA-Seq gene expression data. Thus, the alfalfa transcriptome has not been fully characterized due to the lack of full-length cDNA. In this work, we used PacBio third-generation technology to annotate the sequences of the C cultivar, and analyzed the DEGs in different flower development stages of C and M using Illumina sequencing platform. We obtained 140,995 isoforms, including 513 novel isoforms. After comparison in Swiss-Prot, 204 new isoforms specific to alfalfa, but with unknown functions, were identified and would be useful in future studies (Table S2). In transcriptome studies of populus, maize, and sorghum by single-molecule long-read sequencing, 59,977 (69%), 62,547 (57%) and 11,342 (41%) new isoforms were identified, respectively [36]. Due to species divergence, we only identified 23% new isoforms. However, our data demonstrated that PacBio full-length sequencing could provide a more comprehensive set of isoforms than next-generation sequencing.

Through a genome-based reconstruction strategy, using the *Medicago* genome (*M. truncatula* Mt4.0v2) as a reference, the mapping ratio of the corrected isoforms by PacBio full-length sequencing was 98.52%. Unfortunately, the mapping ratio of the clean reads by RNA-Seq was less than 50% (data not shown). We also compared the match ratio of the isoforms and contigs, from which we found that 99% of the isoforms (33,518) could be matched to known unigenes, indicating that the results of the long-read RNA sequencing were more integrated and accurate.

### Comparison of the genes related to the biosynthesis of flavonoids in different alfalfa materials

Flavonoids are among the most important pigments in the petals of many plants [22, 46]. Anthocyanins are end products of the flavonoid biosynthetic pathway, and generate the widest spectrum of colors, ranging from pale yellow to blue-purple [47]. Our results demonstrated that the color difference between the purple and cream flowers of alfalfa is due to the loss of the flower anthocyanins malvidin and petunidin (Fig. 1). The shift from purple to cream requires a blockage of the anthocyanin biosynthetic pathway, which probably occurs in some reactions before malvidin and petunidin are formed. Therefore, the abundance of the candidate genes was compared in the C and M transcriptomes to identify the key genes of cream color metabolism. Most of the isoforms related to flavonoids synthesis, including *PALs*, *4CLs*, *CHIs*, *DFRs*, *ANSs* and *UFGTs*, showed large-scale higher transcription expression in C with purple flowers than in M with cream flowers, particularly for the first three stages (Fig. 2), indicating that the mutation-induced change in expression by these genes might occur far earlier than the emergence of the phenotype. In the process of flavonoid biosynthesis, CHS catalyzes the first reaction step and help synthesizing the intermediate chalcone, which is extremely important for all classes of flavonoids [48]. So the function restrain of CHS reactions are always accompanied with the elimination of not only anthocyanin biosynthesis, but also other flavonoids compounds [49]. The mutation of a single CHS enzyme led to white flower lines in grape hyacinth [31], petunia [50], *Silene littorea* [33] and arctic mustard flower [51]. Conversely, in our study, we found that *CHSs* showed higher expression in M-S4 than C-S4 (Fig. 10). Interestingly, coumaroyl-CoA can be transformed into isoliquiritigenin (an important product for the isoflavone biosynthesis pathway) by the co-function of CHS and CHR [52, 53]. Upon further data analysis, we found that the expression patterns of *CHRs* were similar to *CHSs* (Fig. 10). We thus speculated that the higher abundance of *CHSs* participated in another branching point in flavonoid biosynthesis, being the intermediates in the production of isoflavone biosynthesis, and CHS and CHR in M-S4 might be crucial for the biosynthesis of isoliquiritigenin.

F3H, F3’H and F3’5’H play critical roles in the flavonoid biosynthetic pathway, they catalyze the hydroxylation of flavonoids including dihydrokaempferol, dihydroquercetin, and dihydromyricetin, which are necessary for anthocyanin biosynthesis [28, 54]. Additionally, the intermediates dihydroflavonols is the main precursor of the coloured anthocyanins production through DFR, and the colourless flavonols production through FLS [55]. So the substrate competition of dihydroflavonols will result in the reverse expression regulation of *FLS* and *DFR*, accompanied by the different accumulation of flavonols and anthocyanin, respectively [55]. In our study, much higher expression of *FLS*/*F3Hs*, *F3’Hs*, and *F3’5’H* was found in most stages of M than C. This was accompanied with the higher expression of *DFR* in C, but at a very low level from S2 to S4 of M (Fig. 10). A similar observation was found by Lou et al. [28], who concluded that *DFR* might be the target gene for the loss of blue pigmentation (delphinidin) in white grape hyacinth. Thus, the higher expression of *FLS*/*F3Hs*, *F3’Hs*, and *F3’5’H* might increase the production of other flavonoid compounds, such as dihydroquercetin, dihydrokaempferol, dihydromyricetin. Myricetin and kaempferol in M, and the down-regulated DFR might partially block the synthesis of anthocyanins, thereby eliminating the process of purple pigmentation.

The purple flower ripening of C suggested that the fundamental transcriptional regulation of the genes from the upstream *PAL* to the end *UFGT* might play important roles in the accumulation of flavonoid intermediates and flower color formation.

### Hub genes related to flower formation were identified by WGCNA

The cream-colored Zhongtian No. 3 alfalfa represents a color mutation, as the purple Defu alfalfa is the wild-type. Understanding the changes in the cream flower phenotype as a mutant of the wild-type could elucidate the mechanisms of the alfalfa flower pigmentation. Any functional loss of key enzymes in the flavonoid biosynthetic pathway could lead to a cream color mutation, including via transcript abundance changes in genes, and branching changes in flavone products [56, 57]. A novel finding from this study was that, by performing WGNCA, we identified floral developmental stage-specific gene modules (Figs. 8 and 9). To this end, 9 *PALs*, 2 *4CLs*, 4 *CHSs*, 3 *CHRs*, *F3’H4*, 2 *DFRs*, and 2 *UFGTs* were highly associated in modules with close relationships to the M4 or M group. They all possessed evident differences in transcript abundance in C and M, indicating their important roles in floral formation variation. It was worth noting that, the above genes were not the genes with the highest expression levels, implying that the high expression genes were not necessary for distinguishing different flower colors [29]. Thus, the WGCNA analyses in this study provided a useful approach for selecting important genes related to the specific phenotypes. Du et al. [58] identified hub genes operating in the seed coat network in the early seed maturation stage by WGCNA analysis. Similar WGCNA analysis was used in golden camellia to identify unigenes correlated with flower color, and CHS, F3H, ANS and FLS were found to play critical roles in regulating the formation of flavonols and anthocyanidins [29].

The 6 hub genes were upstream of the flavonoid biosynthesis pathway, implying that the cream flower pigmentation of M was mainly blocked upstream. The decreased expression of *PAL6*, *PAL9*, and *4CL8*, whether in C or M, is in line with the results in fig [57]. Wang et al. [57] found that the decreased expression of *PALs* and *4CLs* affected the cinnamic acid content in the “Purple Peel” mature fruit peel. We speculated that the decreased expression of *PAL6*, *PAL9*, and *4CL8* might also affect the cinnamic acid content in the petals both in C and M. The elevated expression of *CHSs* in M-S4 might play crucial roles in the biosynthesis of other flavones, such as isoflavone, which is also a crucial factor in the color formation of different flowers in alfalfa.

**Fig. 11:**
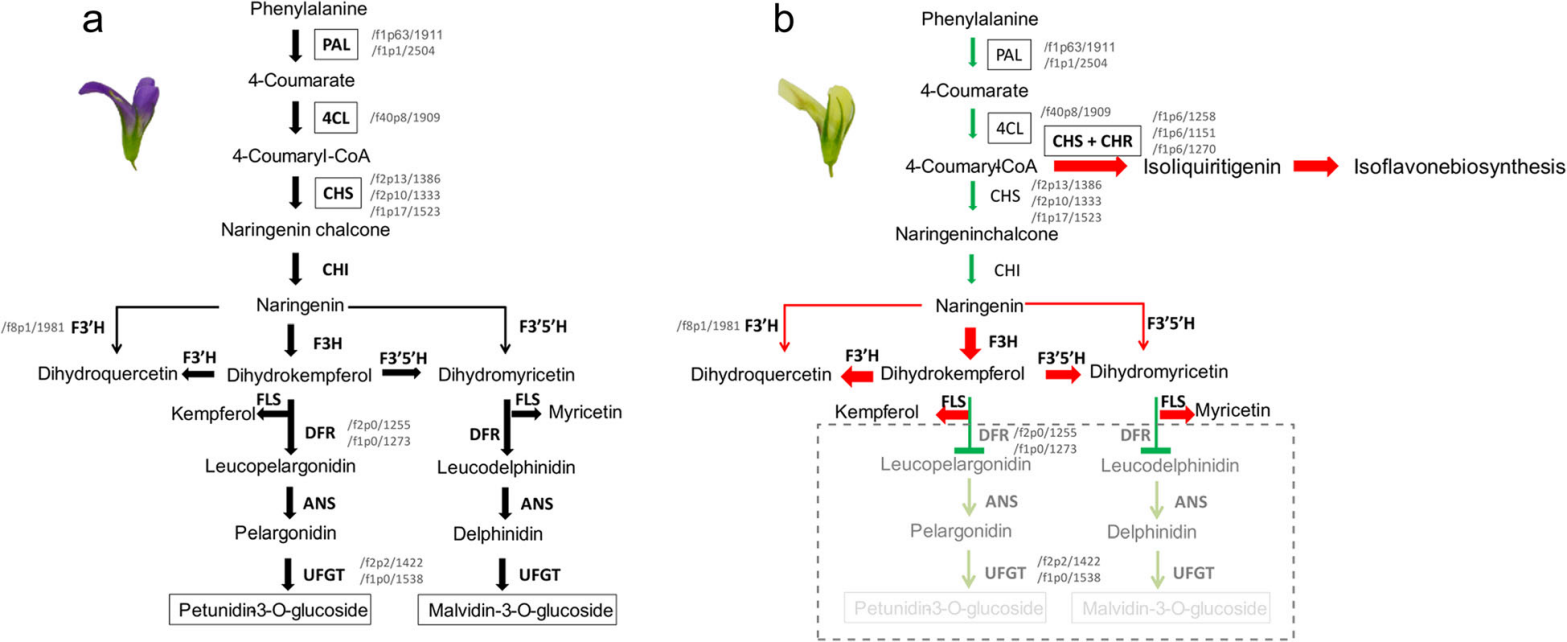
A referred model for the process of anthocyanin synthesis in the purple flowers of C and cream flowers of M. The crucial isoform IDs are indicated at the side of each gene. Upstream of M, *PAL* and *4CL* are suppressed, and an increasing branch of isoflavone biosynthesis regulated by *CHS* and *CHR* is dominant. Furthermore, the up-regulation of *F3H*/*FLS*, *F3’H*, and *F3’5’H* causes an increase in other flavonoid compounds, such as myricetin and kaempferol, further reducing the anthocyanin synthesis. Finally, the low expression level of *DFR* accompanied with the low abundance of *UFGT* might disrupt the anthocyanin synthesis, leading to the formation of the cream color

Based on the above results, different flavonoid biosynthesis pathways in purple- and cream-colored alfalfa were inferred (Fig. 11). Briefly, compared to C, the flavonoid biosynthesis of M is blocked upstream, by *PAL* and *4CL*, following which a branch of isoflavone biosynthesis regulated by *CHS* and *CHR* is dominant, completing the anthocyanin synthesis pathway. Additionally, the up-regulation of *F3H/FLS*, *F3’H*, and *F3’5’H* causes an increase in other flavonoid compounds, such as myricetin and kaempferol, further reducing anthocyanin synthesis. Finally, the low expression level of *DFR* accompanied with the low abundance of *UFGT* might disrupt the anthocyanin synthesis, leading to the formation of the cream color.

## Conclusions

The mechanisms of anthocyanin and flavonoid pathways in the purple flower of Defu and cream flower of Zhongtian No. 3 were analyzed by HPLC, transcriptome analysis and RT-qPCR. Malvidin and petunidin glycoside derivatives were the major anthocyanins in the flowers of Defu, which were lacking in the flowers of Zhongtian No. 3. The PacBio long-read RNA sequencing was more integrated and accurate than RNA-Seq. A new hypothesis is proposed for the lack of purple phenotype in the alfalfa flowers, a series of candidate genes might be co-functioned through flavonoid biosynthesis blocking, the competition of other flavonoid compounds formation, anthocyanin synthesis blocking, and so on. Further research is required to fully elucidate these processes.

## Supplementary information

**Additional file 1: Figure S1.** The significantly enriched KEGG pathway of DEGs between M-S4 and C-S4.

**Additional file 2: Table S1.** Primers for the RT-qPCR.

**Additional file 3: Table S2.** Isoforms statistics of the 513 new isoforms. A total of 309 new isoforms were annotated in the Swiss-Prot database, and the remaining 204 isoforms were unannotated.

**Additional file 4: Table S3.** Isoforms ID of the genes on the heatmap related to the flavonoid synthesis.

**Additional file 5: Table S4.** Enriched module information in all the stages of M, specifically M-S4. The module of skyblue3 displays a close relationship with M-S4, and the modules of bisque4 and turquoise exhibit a close relationship with M. The enriched pathways related to flower color formation of each module are summarized.

## Data Availability

All raw sequence data have been submitted to the Sequence Read Archive (SRA) database under accession number PRJNA565675. The addresses are as follows: https://submit.ncbi.nlm.nih.gov/subs/sra.

## Abbreviations

*4CL*: 4-coumarate: coenzyme A ligase
A3SS: Alternative 3ˊ splice sites
A5SS: Alternative 5ˊ splice sites
*ANS*: Anthocyanidin synthase
C: Defu
CCS: Circular consensus sequences
CDS: The putative coding sequences
*CHI*: Chalcone isomerase
*CHR*: Chalcone reductase
*CHS*: Chalcone synthase
*DFR*: Dihydroflavonol 4-reductase
DGE: Digital gene expression
ES: Exon skipping/inclusion
*F3′5′H*: Flavonoid-3′, 5′-hydroxylase
*F3′H*: Flavonoid 3′-monooxygenase
*F3H*: Flavanone 3-hydroxylase
FDR: False discovery rate
*FLS*: Flavonol synthesis
FPKM: Fragments Per Kilobase of transcript per Million mapped reads
FW: Fresh weight
HPLC: High-performance liquid chromatography analysis
IR: Intron retention
Iso-Seq: Isoform sequencing
KEGG: Kyoto Encyclopedia of Genes and Genomes
M: Zhongtian No. 3
*M. falcata* L.: *Medicago falcata* L.;
*M. lupulina* L.: *Medicago lupulina* L.
*M. sativa* L.: *Medicago sativa* L.
*M. truncatula*: *Medicago truncatula*
*M. varia*: *Medicago varia*
MXE: Mutually exclusive exons
NR: The protein databases of Non-redundant
ORFs: Open reading frames
*PAL*: Phenylalanine ammonia-lyase
PCA: Principal component analysis
RIN: RNA Integrity Number
RNA-Seq: RNA sequencing
RT-qPCR: Real-time quantitative PCR
SEs: Standard errors
TFs: Transcription factors
TO: Topological overlap
*UFGT*: UDP-glucose: flavonoid 3-O-glucosyltransferase
WGCNA: Weighted gene co-expression network analysis
