# Supplementary figures and images for: Identification of the regulatory networks and hub genes controlling alfalfa floral pigmentation variation using RNA-sequencing analysis
Source: BMC Plant Biol. 2020 Mar 12;20:110. doi: 10.1186/s12870-020-2322-9 (PMC7068929; doi:10.1186/s12870-020-2322-9)

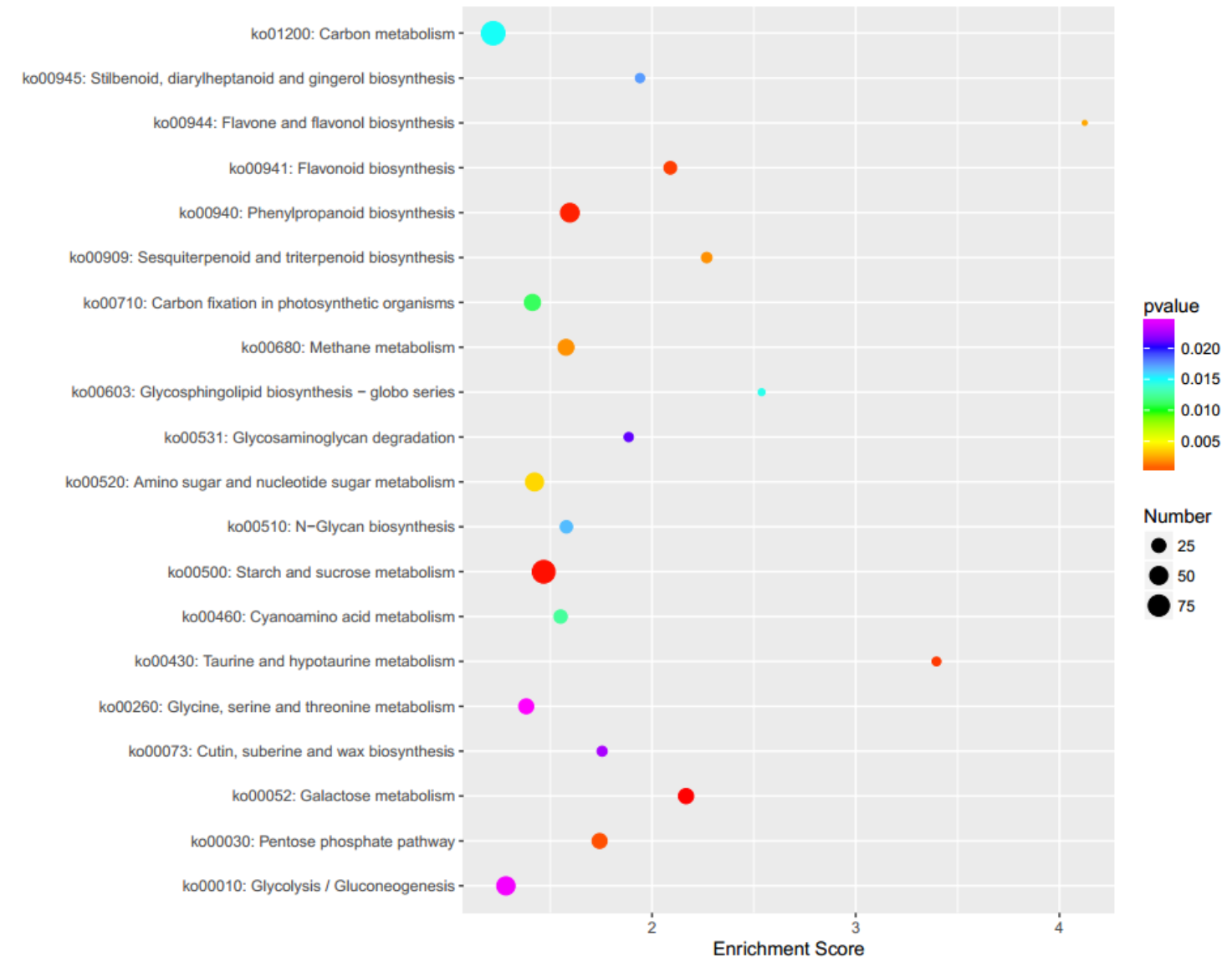

Supplement: Supplementary file 1 — Additional file 1: Figure S1. The significantly enriched KEGG pathway of DEGs between M-S4 and C-S4. [file 12870_2020_2322_MOESM1_ESM.tif]
