# Supplementary material for: Identification of the regulatory networks and hub genes controlling alfalfa floral pigmentation variation using RNA-sequencing analysis
Source: BMC Plant Biol. 2020 Mar 12;20:110. doi: 10.1186/s12870-020-2322-9 (PMC7068929; doi:10.1186/s12870-020-2322-9)
Supplement: Supplementary file 2 — Additional file 2: Table S1. Primers for the RT-qPCR. [file 12870_2020_2322_MOESM2_ESM.docx]

**Supplementary Table S1** Primer sequences for RT-qPCR analysis.

| **Gene** | **Forward primer** | **Reverse primer** | **Product length (bp)** |
| --- | --- | --- | --- |
| PAL6 | CCACACACGGCAACAAGAGCAG | CGAAGCGGTAAACATGGAGTGACG | 137 |
| PAL9 | CACAGCTATGTGCCAGGGAA | ATCTGAGAAACTTGAAATGAACCTT | 192 |
| CHS2 | TTGTTCTGAGGTCACCGCTGTC | TCCATCTCCAAATAGTGCTTGTCC | 88 |
| CHS4 | ACAACACACTTCAACCCTTTGC | GCAGGACCTTCTGCTCTTTGA | 148 |
| CHR1 | CCAGTGGTTGGAATGGGATCAGC | AGGCAGCAGCAGTATCAAAGTGTC | 112 |
| CHR2 | TCCCAACAACAACAACATGGGT | GGCTGATCCCATTCCAACCA | 100 |
| CHR3 | TGCTGCCTATGGCTCAGAAC | GCTCATCTCTAGTGACAAGACCAA | 80 |
| F3’H4 | ACGACACCGTTTTTGCCAAC | TCCGTACGGGCTCCATGATA | 89 |
| DFR1 | GCGACCCAGATAACATAAAGAAGGT | CTACCCTCTTCACCAAGGTCAGC | 100 |
| DFR2 | CTTCGGGTTTCATCGGGTCA | CAAGTTTTCTGGGTCGCGGA | 89 |
| UFGT22 | CACGGCCTCAAACTTCCTCA | ACAAGTTGCTGGTGTTGTGAAC | 88 |
| UFGT23 | TATGTGCTGGGGTGCCAATG | CCACTCCAACCATCACGCAA | 103 |
| Rer1 | GCCTTCTGATGGTGGACCT | GGCCAGAAGACAGGAACATC | 165 |
