# Supplementary material for: Identification of the regulatory networks and hub genes controlling alfalfa floral pigmentation variation using RNA-sequencing analysis
Source: BMC Plant Biol. 2020 Mar 12;20:110. doi: 10.1186/s12870-020-2322-9 (PMC7068929; doi:10.1186/s12870-020-2322-9)
Supplement: Supplementary file 4 — Additional file 4: Table S3. Isoforms ID of the genes on the heatmap related to the flavonoid synthesis. [file 12870_2020_2322_MOESM4_ESM.docx]

Supplementary Table S3 Isoforms ID of the genes on the heatmap related to the flavonoid synthesis.

| Gene name | Gene Id |
| --- | --- |
| PAL1 | PB.11849.10\|chr7:40942885-40960253(+)\|i2_LQ_samplef2cfa8\|c97668/f1p0/2837 |
| PAL2 | PB.11849.12\|chr7:40942885-40959874(+)\|i2_LQ_samplef2cfa8\|c71112/f1p0/2392 |
| PAL3 | PB.11849.14\|chr7:40942885-40960512(+)\|i3_LQ_samplef2cfa8\|c5006/f1p3/3081 |
| PAL4 | PB.11849.16\|chr7:40942887-40959833(+)\|i2_LQ_samplef2cfa8\|c94554/f1p1/2514 |
| PAL5 | PB.11849.17\|chr7:40943081-40945873(+)\|i2_LQ_samplef2cfa8\|c136140/f1p0/2171 |
| PAL6 | PB.11849.2\|chr7:40942885-40959392(+)\|i1_LQ_samplef2cfa8\|c131710/f1p63/1911 |
| PAL7 | PB.11849.3\|chr7:40942885-40959926(+)\|i2_HQ_samplef2cfa8\|c113826/f130p0/2449 |
| PAL8 | PB.11849.4\|chr7:40942885-40945992(+)\|i2_LQ_samplef2cfa8\|c4595/f1p3/2499 |
| PAL9 | PB.11849.8\|chr7:40942885-40959918(+)\|i2_LQ_samplef2cfa8\|c27489/f1p1/2504 |
| PAL10 | PB.1618.1\|chr1:42608832-42615637(-)\|i2_LQ_samplef2cfa8\|c127052/f3p2/2594 |
| PAL11 | PB.916.1\|chr1:28183235-28188005(-)\|i2_LQ_samplef2cfa8\|c115382/f1p0/2672 |
| PAL12 | PB.916.2\|chr1:28183331-28187899(-)\|i4_LQ_samplef2cfa8\|c1792/f1p0/4422 |
| PAL13 | PB.916.3\|chr1:28183352-28187904(-)\|i2_LQ_samplef2cfa8\|c22425/f1p2/2614 |
| PAL14 | PB.916.5\|chr1:28183457-28187899(-)\|i2_LQ_samplef2cfa8\|c133138/f1p0/2426 |
| PAL15 | PB.916.6\|chr1:28183562-28187892(-)\|i2_LQ_samplef2cfa8\|c20025/f1p0/2309 |
| PAL16 | PB.9841.1\|chr5:43212802-43217702(-)\|i2_HQ_samplef2cfa8\|c6525/f8p0/2317 |
| 4CL1 | PB.1115.1\|chr1:32460145-32466471(+)\|i2_LQ_samplef2cfa8\|c15805/f1p0/2033 |
| 4CL2 | PB.1115.3\|chr1:32460171-32466171(+)\|i1_LQ_samplef2cfa8\|c211145/f1p0/1638 |
| 4CL3 | PB.1545.3\|chr1:40755033-40757667(+)\|i2_HQ_samplef2cfa8\|c76958/f2p1/2016 |
| 4CL4 | PB.3579.1\|chr2:40805509-40810273(-)\|i2_LQ_samplef2cfa8\|c51057/f2p1/2017 |
| 4CL5 | PB.3579.3\|chr2:40805636-40810321(-)\|i1_LQ_samplef2cfa8\|c85586/f5p1/1962 |
| 4CL6 | PB.3829.1\|chr2:45520846-45525716(+)\|i2_LQ_samplef2cfa8\|c90039/f2p0/2157 |
| 4CL7 | PB.3829.2\|chr2:45520846-45525666(+)\|i2_LQ_samplef2cfa8\|c28586/f1p0/2193 |
| 4CL8 | PB.3829.3\|chr2:45520846-45525682(+)\|i2_LQ_samplef2cfa8\|c5977/f2p2/2373 |
| 4CL9 | PB.3830.2\|chr2:45521006-45525684(-)\|i2_LQ_samplef2cfa8\|c25236/f1p0/2342 |
| 4CL10 | PB.405.2\|chr1:8532184-8600630(-)\|i2_HQ_samplef2cfa8\|c114316/f5p1/2071 |
| 4CL11 | PB.405.3\|chr1:8532200-8600514(-)\|i2_LQ_samplef2cfa8\|c73521/f1p1/2080 |
| 4CL12 | PB.405.4\|chr1:8532291-8600510(-)\|i1_LQ_samplef2cfa8\|c131814/f1p11/1834 |
| 4CL13 | PB.4081.1\|chr3:12041744-12049881(-)\|i1_LQ_samplef2cfa8\|c164774/f1p0/1978 |
| 4CL14 | PB.4253.1\|chr3:23869958-23873008(+)\|i1_LQ_samplef2cfa8\|c42061/f1p0/1849 |
| 4CL15 | PB.4636.1\|chr3:34017547-34032101(+)\|i1_LQ_samplef2cfa8\|c13059/f4p2/1838 |
| 4CL16 | PB.4636.2\|chr3:34027339-34032100(+)\|i1_LQ_samplef2cfa8\|c12554/f4p2/1849 |
| 4CL17 | PB.4636.3\|chr3:34027344-34032198(+)\|i1_LQ_samplef2cfa8\|c36558/f1p2/1923 |
| 4CL18 | PB.5838.1\|chr4:349590-353192(+)\|i1_HQ_samplef2cfa8\|c237238/f40p8/1909 |
| 4CL19 | PB.6818.1\|chr4:29911458-29915244(+)\|i2_LQ_samplef2cfa8\|c2798/f2p1/2222 |
| 4CL20 | PB.6818.2\|chr4:29911759-29914980(+)\|i1_LQ_samplef2cfa8\|c7582/f1p2/1675 |
| 4CL21 | PB.8087.1\|chr4:53452938-53459625(+)\|i2_LQ_samplef2cfa8\|c39369/f1p0/2201 |
| 4CL22 | PB.8087.5\|chr4:53453111-53459491(+)\|i1_LQ_samplef2cfa8\|c10179/f1p0/1987 |
| 4CL23 | PB.8345.1\|chr5:1444085-1450165(+)\|i2_LQ_samplef2cfa8\|c64507/f1p12/2098 |
| 4CL24 | PB.8345.2\|chr5:1444137-1450502(+)\|i2_HQ_samplef2cfa8\|c34927/f4p0/2074 |
| 4CL25 | PB.8861.1\|chr5:11438244-11442734(-)\|i2_LQ_samplef2cfa8\|c114526/f10p1/2126 |
| 4CL26 | PB.8862.1\|chr5:11450368-11453308(-)\|i2_LQ_samplef2cfa8\|c51606/f1p1/2061 |
| 4CL27 | PB.8862.2\|chr5:11450502-11453421(-)\|i2_LQ_samplef2cfa8\|c80656/f1p1/2020 |
| CHS1 | PB.10727.1\|chr7:5288756-5290374(-)\|i1_LQ_samplef2cfa8\|c23258/f3p20/1452 |
| CHS2 | PB.10728.1\|chr7:5301940-5316126(+)\|i1_LQ_samplef2cfa8\|c190118/f2p13/1386 |
| CHS3 | PB.10728.2\|chr7:5301941-5316113(+)\|i1_LQ_samplef2cfa8\|c25700/f1p17/1378 |
| CHS4 | PB.10728.3\|chr7:5301944-5316192(+)\|i1_HQ_samplef2cfa8\|c217277/f2p10/1333 |
| CHS5 | PB.11432.1\|chr7:32530009-32532163(-)\|i1_LQ_samplef2cfa8\|c2632/f6p8/1586 |
| CHS6 | PB.11432.2\|chr7:32530141-32532156(-)\|i1_LQ_samplef2cfa8\|c130915/f1p7/1421 |
| CHS7 | PB.13346.1\|chr8:35250318-35251835(+)\|i1_HQ_samplef2cfa8\|c6550/f4p8/1391 |
| CHS8 | PB.1696.1\|chr1:44128070-44142309(+)\|i1_LQ_samplef2cfa8\|c11658/f1p17/1523 |
| CHS9 | PB.1696.2\|chr1:44128070-44142204(+)\|i1_LQ_samplef2cfa8\|c62837/f2p16/1411 |
| CHS10 | PB.1696.3\|chr1:44128070-44133768(+)\|i1_LQ_samplef2cfa8\|c66583/f6p22/1388 |
| CHS11 | PB.4839.1\|chr3:37858378-37860058(-)\|i1_HQ_samplef2cfa8\|c237312/f11p5/1544 |
| CHS12 | PB.4840.1\|chr3:37862308-37864216(-)\|i1_LQ_samplef2cfa8\|c23807/f1p6/1848 |
| CHS13 | PB.8347.1\|chr5:1477527-1487854(+)\|i1_LQ_samplef2cfa8\|c240015/f3p13/1389 |
| CHS14 | PB.8347.2\|chr5:1481568-1493170(+)\|i1_LQ_samplef2cfa8\|c47649/f4p19/1451 |
| CHS15 | PB.8348.1\|chr5:1481568-1487857(-)\|i1_LQ_samplef2cfa8\|c41994/f1p4/1609 |
| CHI1 | PB.2128.1\|chr1:52317616-52318844(+)\|i1_LQ_samplef2cfa8\|c282609/f1p2/1005 |
| CHI2 | PB.2129.1\|chr1:52333401-52334681(-)\|i1_LQ_samplef2cfa8\|c274650/f1p2/1012 |
| CHI3 | PB.5140.1\|chr3:42940682-42942411(+)\|i0_HQ_samplef2cfa8\|c18729/f23p0/931 |
| FLS1 | PB.4570.1\|chr3:32763532-32765066(-)\|i1_LQ_samplef2cfa8\|c13462/f1p1/1293 |
| FLS2 | PB.4570.2\|chr3:32763692-32765066(-)\|i1_LQ_samplef2cfa8\|c224350/f1p1/1139 |
| FLS3 | PB.9227.1\|chr5:24398458-24401646(+)\|i1_LQ_samplef2cfa8\|c64479/f2p4/1341 |
| F3'H1 | PB.4017.1\|chr3:7675008-7683447(+)\|i1_LQ_samplef2cfa8\|c151510/f3p1/1749 |
| F3'H2 | PB.7477.1\|chr4:42376015-42378949(-)\|i2_HQ_samplef2cfa8\|c2138/f4p0/2119 |
| F3'H3 | PB.7477.2\|chr4:42376257-42378956(-)\|i1_HQ_samplef2cfa8\|c5495/f2p1/1860 |
| F3'H4 | PB.7478.2\|chr4:42392721-42394930(-)\|i1_HQ_samplef2cfa8\|c1984/f8p1/1981 |
| F3'H5 | PB.7659.3\|chr4:45477181-45481740(+)\|i1_HQ_samplef2cfa8\|c237617/f10p1/1941 |
| F3'5'H | PB.4084.1\|chr3:12278062-12281867(-)\|i2_HQ_samplef2cfa8\|c102456/f6p1/2042 |
| AOMT | PB.7235.1\|chr4:37833577-37835915(-)\|i0_LQ_samplef2cfa8\|c20391/f1p0/826 |
| DFR1 | PB.339.2\|chr1:7156508-7160534(-)\|i1_HQ_samplef2cfa8\|c21297/f2p0/1255 |
| DFR2 | PB.340.1\|chr1:7164081-7167125(-)\|i1_LQ_samplef2cfa8\|c4738/f1p0/1273 |
| DFR3 | PB.5357.1\|chr3:46716957-46719394(+)\|i1_HQ_samplef2cfa8\|c124277/f3p0/1346 |
| DFR4 | PB.5357.2\|chr3:46716964-46719215(+)\|i1_LQ_samplef2cfa8\|c21108/f1p0/1160 |
| DFR5 | PB.6794.1\|chr4:29554870-29557289(+)\|i1_LQ_samplef2cfa8\|c23477/f1p3/1343 |
| ANS1 | PB.13205.1\|chr8:31320120-31332067(-)\|i1_LQ_samplef2cfa8\|c36588/f4p2/1304 |
| ANS2 | PB.8440.1\|chr5:3196923-3199377(+)\|i1_LQ_samplef2cfa8\|c7773/f5p0/1359 |
| ANS3 | PB.8440.2\|chr5:3196930-3199422(+)\|i1_LQ_samplef2cfa8\|c42762/f1p0/1409 |
| ANS4 | PB.8440.3\|chr5:3196932-3199376(+)\|i1_HQ_samplef2cfa8\|c40423/f4p0/1334 |
| UFGT1 | PB.2783.1\|chr2:13439523-13441448(-)\|i1_LQ_samplef2cfa8\|c56802/f2p3/1919 |
| UFGT2 | PB.2783.2\|chr2:13439659-13441448(-)\|i1_LQ_samplef2cfa8\|c193327/f2p3/1777 |
| UFGT3 | PB.2785.2\|chr2:13447268-13449570(-)\|i2_LQ_samplef2cfa8\|c46030/f1p11/2300 |
| UFGT4 | PB.2785.4\|chr2:13447783-13462620(-)\|i1_LQ_samplef2cfa8\|c2872/f4p4/1845 |
| UFGT5 | PB.6187.1\|chr4:11023032-11024681(+)\|i1_LQ_samplef2cfa8\|c235897/f22p6/1648 |
| UFGT6 | PB.6187.2\|chr4:11023036-11024406(+)\|i1_HQ_samplef2cfa8\|c236507/f2p6/1372 |
| UFGT7 | PB.6996.2\|chr4:33396182-33397904(-)\|i1_LQ_samplef2cfa8\|c83058/f3p0/1722 |
| UFGT8 | PB.6996.3\|chr4:33396328-33397904(-)\|i1_LQ_samplef2cfa8\|c17555/f5p0/1581 |
| UFGT9 | PB.7835.1\|chr4:48911254-48912915(+)\|i1_LQ_samplef2cfa8\|c38706/f2p3/1642 |
| UFGT10 | PB.7836.1\|chr4:48957558-48959343(-)\|i1_LQ_samplef2cfa8\|c160481/f1p10/1798 |
| UFGT11 | PB.7837.1\|chr4:48964460-48966512(-)\|i2_LQ_samplef2cfa8\|c107009/f2p0/2048 |
| UFGT12 | PB.8900.1\|chr5:12539903-12541877(-)\|i1_LQ_samplef2cfa8\|c87990/f2p3/2002 |
| UFGT13 | PB.9307.2\|chr5:28384706-28386580(-)\|i1_HQ_samplef2cfa8\|c148826/f12p3/1929 |
| UFGT14 | PB.9307.3\|chr5:28384730-28386487(-)\|i1_HQ_samplef2cfa8\|c19229/f2p3/1754 |
| UFGT15 | PB.9307.4\|chr5:28384934-28386566(-)\|i1_LQ_samplef2cfa8\|c38330/f1p3/1638 |
| UFGT16 | PB.9675.2\|chr5:39454603-39461728(+)\|i1_LQ_samplef2cfa8\|c66276/f2p6/1511 |
| UFGT17 | PB.9675.3\|chr5:39459972-39461695(+)\|i1_LQ_samplef2cfa8\|c211399/f1p6/1702 |
| UFGT18 | PB.9675.5\|chr5:39486334-39488424(+)\|i2_LQ_samplef2cfa8\|c81398/f1p7/2012 |
| UFGT19 | PB.9675.6\|chr5:39486362-39487950(+)\|i1_LQ_samplef2cfa8\|c56216/f5p5/1571 |
| UFGT20 | PB.9676.5\|chr5:39463191-39464819(-)\|i1_LQ_samplef2cfa8\|c129722/f1p5/1675 |
| UFGT21 | PB.9848.1\|chr5:43311230-43312833(-)\|i1_HQ_samplef2cfa8\|c23063/f8p5/1607 |
