# Supplementary material for: Identification of the regulatory networks and hub genes controlling alfalfa floral pigmentation variation using RNA-sequencing analysis
Source: BMC Plant Biol. 2020 Mar 12;20:110. doi: 10.1186/s12870-020-2322-9 (PMC7068929; doi:10.1186/s12870-020-2322-9)
Supplement: Supplementary file 5 — Additional file 5: Table S4. Enriched module information in all the stages of M, specifically M-S4. The module of skyblue3 displays a close relationship with M-S4, and the modules of bisque4 and turquoise exhibit a close relationship with M. The enriched pathways related to flower color formation of each module are summarized. [file 12870_2020_2322_MOESM5_ESM.docx]

Supplementary Table S4 Enriched module information in all the stages of M, specifically M-S4. The module of skyblue3 displayed a close relationship with M-S4, and the modules of bisque4 and turquoise exhibited a close relationship with M. The enriched pathways related to flower color formation of each module are summarized.

| Module | Terms ID | Enriched pathways related to color formation | List hit | *P*-value |
| --- | --- | --- | --- | --- |
| skyblue3 | ko00941 | flavonoid biosynthesis | 8 | 0.000000 |
|  | ko00940 | phenylpropanoid biosynthesis | 9 | 0.000622 |
|  | GO:0009813 | flavonoid biosynthetic process | 10 | 0.000000 |
|  | GO:0009715 | chalcone biosynthetic process | 3 | 0.000008 |
|  | GO:0016210 | naringenin-chalcone synthase activity | 4 | 0.000018 |
| bisque4 | ko00940 | phenylpropanoid biosynthesis | 32 | 0.000000 |
|  | GO:0009800 | cinnamic acid biosynthetic process | 9 | 0.000000 |
|  | GO:0006559 | L-phenylalanine catabolic process | 9 | 0.000000 |
| turquoise | GO:0047172 | shikimate O-hydroxycinnamoyltransferase activity | 5 | 0.00173 |
|  | GO:0045552 | dihydrokaempferol 4-reductase activity | 3 | 0.002882 |
|  | GO:0080046 | quercetin 4'-O-glucosyltransferase activity | 4 | 0.005734 |
